# Between willingness and practice: a nationwide survey of 1,334 German patient organization members on user involvement in digital service development

**DOI:** 10.3389/fdgth.2025.1591981

**Published:** 2025-06-06

**Authors:** Simon Wallraf, Marie-Luise Dierks, Sabine Wöhlke, Cosima John, Jonas Lander

**Affiliations:** ^1^ Institute for Epidemiology, Social Medicine and Health Systems Research, Hannover Medical School, Hanover, Germany; ^2^ Department of Health Sciences, Hamburg University of Applied Sciences, Hamburg, Germany

**Keywords:** digital health, user involvement, patient involvement, patient organizations, digital service development, volunteering, digital literacy

## Abstract

**Introduction:**

As the digital transformation of healthcare progresses, key actors such as patient organizations (POs) are adapting their activities and services to digital formats. This study explores how PO members are involved in developing digital services, focusing on their general willingness, actual involvement, and associated factors.

**Methods:**

A nationwide online survey was conducted among members of German POs from August to November 2023. Participants were recruited through 300 national POs. Data analysis included descriptive statistics and multivariate logistic regression analyses to examine potential predictors of three involvement variables.

**Results:**

Of the 1,334 participants, the majority were female (67.2%) and aged ≥50 years (65.6%). While only 22.4% of respondents had been approached by their PO to contribute to digital services — most commonly to PO websites, focusing primarily on content development — 81.2% emphasized the importance of member involvement, and nearly half (48%) expressed willingness to engage. Members volunteering within their PO were significantly more likely than non-volunteers to express willingness (OR = 2.905, 95% CI: 2.163–3.901, *p* < 0.001) and to be approached by their PO (OR = 5.227, 95% CI: 3.765–7.256, *p* < 0.001). Additionally, members not engaged in volunteer roles were significantly less likely to agree to such a request (OR = 0.076, 95% CI: 0.032–0.181, *p* < 0.001). Members with poor self-rated digital skills were significantly less likely to express willingness (OR = 0.235, 95% CI: 0.135–0.407, *p* < 0.001) or to be involved (OR = 0.070, 95% CI: 0.016–0.300, *p* < 0.001) than those with strong digital skills. Other factors, such as age, gender, educational attainment, and membership duration, were significantly associated with specific aspects of involvement.

**Conclusion:**

The findings highlight a notable gap between the broad willingness of PO members to engage in digital service development and the limited actual involvement opportunities currently provided by POs. This suggests that structured involvement processes may not yet be fully established, leaving substantial potential untapped. To gain a more comprehensive understanding, future research should explore POs’ perspectives on the feasibility of member involvement, as well as structural and organizational factors that shape these opportunities.

## 1 Introduction

Patient organizations (POs) play a vital role in healthcare systems worldwide. They offer support and advocate for individuals affected by, for example, a chronic disease or a disability, as well as their relatives (1–3). Their support services include individual counseling, support groups, and health education (1, 4, 5). Many POs also represent the interests and needs of affected individuals within the healthcare sector, for example by influencing health policy or contributing to health research (6–10).

In Germany, POs vary not only in how broadly they engage in such activities, but also in their organizational structure. According to a study by Kofahl et al. (9), nearly half of German POs (47%) operate entirely on a voluntary basis, while others have the capacity to employ full-time staff — ranging from a single employee (17%) to five or more (15%). Membership sizes also vary widely, from small organizations with only a few individuals to large organizations with over 50,000 members. About two-thirds of members are directly affected by a health condition, while about one-quarter are family members. As a result, many German POs place a strong focus on directly supporting and empowering their members — for instance, through efforts to improve health literacy, strengthen self—management skills, and provide emotional support (9).

As POs adapt to digitalization in the German healthcare sector and beyond, their support efforts are expanding and evolving as they increasingly integrate digital tools into their services, activities, and internal structures. While most have established websites and use basic digital communication tools, some also engage with members via social media or online forums (11, 12). A few POs have implemented more complex digital solutions, such as their own mobile applications or digital patient registries designed to collect health data for research purposes (11–14). While adopting digital technologies can improve support services for PO members, it also presents challenges, such as addressing members' varying digital literacy levels or ensuring that new digital services meet their specific needs (5, 12). Effectively addressing such challenges is not unique to POs but reflects a broader principle in the design and implementation of digital health technologies and services: ensuring that they are beneficial, user-friendly, widely accepted and trusted by potential end users to maximize their impact and adoption (15–18). This can be achieved by actively involving those directly affected or expected to benefit, such as PO members, and incorporating their needs and interests into the development process (15, 16, 18–20).

In the context of digital health technology development, various terms such as *user-centered design*, *participatory design*, *co-design*, and *co-creation* are used to describe the involvement of users. However, these terms are often used interchangeably across disciplines, with recent reviews pointing to the need for more consistent terminology and clearer reporting of involvement practices (18, 19). In response to this, there have been efforts to differentiate involvement approaches based on the extent of actual user engagement — ranging from passive roles such as providing feedback, to active collaboration where users and designers work together as equal partners, and even to user-driven innovation, in which users lead the development process themselves and make key design decisions (21). Research suggests that, in current practice, user involvement typically reflects rather passive forms of engagement, with users predominantly contributing by providing input through interviews, surveys, or focus groups, or by giving feedback on prototypes (18, 19, 22, 23). To ensure clarity, we use the term user involvement as an umbrella concept throughout this paper.

Although user involvement is increasingly discussed and applied in digital health technology development, little is known about its adoption by German POs. It remains unclear whether and to what extent PO members are involved in the development of digital services or how willing they are to engage in such efforts. Previous research, for instance, has shown that many POs generally struggle to mobilize their members for tasks (9). Whether similar challenges arise in the context of digital service development remains unknown.

To address these research gaps, we explore the perspectives of PO members through the following research questions:

1) How have PO members been involved in the development of digital PO services so far and what were their experiences with this?
2) To what extent are PO members willing to engage in the development of digital PO services, and what factors influence their willingness?

Understanding members' perspectives is essential for aligning PO digitalization efforts and involvement activities with their preferences and abilities, enabling the development of digital services that meet their needs.

## 2 Methods

### 2.1 Study design and population

We conducted a standardized nationwide online survey to capture the diversity of German POs and reflect different perspectives and experiences of members. The study is part of the interdisciplinary research consortium PANDORA (*Patient-centered Digitalization: An Ethical Analysis of the Role of Patient Organizations as Actors in the Context of Digitalization in Health-related Research and Care;* https://www.pandora-forscht.de). The consortium focuses on ethical and social aspects of digitalization in the context of POs, addressing both the digital transformation within these organizations and their role in shaping the digitalization of healthcare and health-related research. Eligible participants were adult PO members (≥18 years) in Germany. The survey was conducted from August 8 to November 20, 2023.

### 2.2 Survey development and measures

In the absence of validated instruments on this topic, we conducted a purposive literature review and drew on findings from our earlier qualitative interview study to develop a questionnaire (12). The questionnaire included seven content sections and one section for sociodemographic data. As this questionnaire was developed in the context of the PANDORA research consortium, it addressed various aspects of PO digitalization, such as members' attitudes towards POs collaborating with external actors, or the willingness of PO members to donate digital health data to a PO patient registry (see Supplementary Material A for the full questionnaire translated into English).

Regarding member involvement in the digital transformation of POs, we focused on the development and design of digital services, as members are their primary users. To examine their involvement in such processes, we considered two key aspects:

(1) **Previous experiences with involvement:** This theme explored the extent to which actual involvement processes have taken place. It focused on aspects such as whether members had been approached by their PO to contribute to the development of digital services, whether they agreed to get involved, how the involvement process unfolded, and how they perceived their experiences.
(2) **Willingness to get involved:** This theme explored the general willingness of members to engage in the development of a digital PO service, including the specific forms of involvement they would consider, and the conditions or requirements that would influence their willingness.

To provide a common reference point for participants, a hypothetical scenario preceded the section of the questionnaire that addressed member involvement. Participants were asked to imagine that their PO was creating a new digital service for its members, such as redesigning its website or developing a mobile app. This service would provide access to health information, allow users to upload personal data, and include features for communication with other members, among other functions. In this scenario, members were approached by their PO to get involved in the development process of this new service. The scenario was designed to ensure a consistent context for all participants.

The questionnaire was developed and administered by the project team based at the Hannover Medical School using SoSci Survey. The initial draft was then refined based on feedback from our project partners at the Hamburg University of Applied Sciences (HAW Hamburg) and University Medical Center Göttingen (UMG), as well as input from our project advisory board of PO representatives, which was gathered through a group discussion [see Supplementary Material B (B1) for details on the advisory board composition]. A pre-test was conducted to assess comprehensibility, relevance, and usability, involving members and representatives of POs.

### 2.3 Recruitment and data collection

We compiled a list of *n* = 300 national-level POs using the membership lists from two umbrella organizations and a database maintained by a national contact and information center for self-help in Germany [see Supplementary Material B (B2 and B3) for further details]. Starting on August 8, 2023, we began sending study invitations, including an information flyer, to all identified POs, asking them to forward the invitation and survey link to their members. Invitations were sent to the general email addresses provided on the organizations' websites and, where appropriate, additionally to board members, managing directors, or named contact persons. Additional recruitment efforts included two reminders, sent in October and November 2023, distribution of the invitation by project advisory board members, and direct outreach by the umbrella organizations to their member organizations. Data collection was completed on November 20, 2023.

### 2.4 Data analysis

Statistical analysis was performed using IBM SPSS version 29 (IBM Corp., Armonk, NY). The dataset was checked for plausibility, completeness, and accuracy. Only respondents who completed the questionnaire were included in the analyses, while those who dropped out were excluded. Missing data, including “no answer” choices or non-responses, were identified across all variables in fully completed questionnaires. For the descriptive analysis of all variables included in this study — variables related to involvement, sociodemographics, and other participant characteristics — absolute and relative frequencies were calculated, as all variables were categorical.

Then, cross-tabulations were created to examine associations between respondent characteristics — such as sociodemographics, self-rated digital skills (reflecting one's ability to use digital technologies and services), and membership information — and involvement outcomes, including general willingness, readiness for different forms of involvement, being approached by the PO, and agreeing to get involved. To assess the statistical significance of associations, either Chi-square tests or Fisher-Freeman-Halton exact tests were applied, depending on the distribution of expected cell counts. To account for multiple comparisons, Bonferroni correction was applied to adjust *p*-values.

Building on these analyses, multivariate logistic regression analyses were conducted to identify potential predictors for three key aspects of involvement: willingness to get involved, being approached by the PO to get involved, and agreeing to it. These variables were chosen because they represent essential aspects of member engagement — namely, the extent to which involvement has already been facilitated and the general readiness of members to get involved. Independent variables selected for the cross-tabulation analyses were included in the multivariate logistic regression models, regardless of whether their associations with the outcome variables were statistically significant. This approach was chosen to account for potential confounding effects and to ensure that even variables with weaker individual associations were considered, allowing for the detection of effects that may only become apparent when other variables are controlled for. Multicollinearity was assessed using variance inflation factors (VIF) and tolerance values, with all variables remaining within acceptable limits (VIF <5, tolerance >0.1). The commonly recommended minimum of ten outcome events per predictor parameter was checked and fulfilled in all models. Categories with fewer than ten cases in one of the outcome variable's response categories (‘yes' or ‘no’) were generally merged or excluded to prevent estimation instability; exceptions were made where conceptual distinctions between categories were deemed important. These decisions are transparently reported in the footnotes to Tables 2–4 in the manuscript. The reference category for each independent variable was set to the category with the highest number of cases, either the first or last category, to ensure stable estimates. The goodness of fit for the logistic regression models was assessed using the Omnibus Test of Model Coefficients, Nagelkerke R^2^, and the Hosmer-Lemeshow test. Results are presented as odds ratios (ORs) with 95% confidence intervals (CIs), using a significance level of *p* < 0.05.

For all analyses other than descriptive statistics, a complete case approach was applied, with cases excluded listwise depending on the variables used in each analysis. Some variables contained larger proportions of missing data due to preceding filter questions that excluded certain subgroups based on specific responses as part of the questionnaire design (for details on filtering mechanisms, see the questionnaire in Supplementary Material A). These were included as independent variables in the cross-tabulation analyses. However, in multivariate logistic regression models, independent variables with substantial missing data due to filtering were excluded to maintain statistical model stability. The dependent variable “agreeing to get involved” was retained despite substantial missing data, as the filtering was specifically designed to target a relevant subgroup of participants who had previously reported being approached by their PO.

### 2.5 Ethics statement

This study was approved by the Ethics Committee of the Hannover Medical School (Approval No.: 10901_BO_K_2023) and conducted in accordance with the Declaration of Helsinki. Informed consent was obtained from all participants prior to participation. They were informed about the purpose of the study, the voluntary nature of participation, their right to withdraw at any time without consequence, and the handling of their data in accordance with the General Data Protection Regulation (GDPR).

## 3 Results

### 3.1 Participant characteristics

A total of 1,334 PO members participated in the survey (see Table 1 for detailed participant characteristics). The majority were women (67.2%, *n* = 896), with men representing 30.7% (*n* = 409). Participants aged 50–64 years constituted the largest age group (41.6%, *n* = 555), followed by those aged 65 years and older (24%, *n* = 320). Nearly half had a college or university degree (46.2%, *n* = 616). Most participants indicated having a chronic disease or disability (75.3%, *n* = 1,004); 57.4% (*n* = 776) indicated multiple conditions. More than half were long-term members of their PO, including 36.9% (*n* = 492) who had been members for more than ten years. The majority rated their own ability to use digital technologies and services (self-rated digital skills) as very good (33.7%, *n* = 449) or rather good (53.9%, *n* = 719). One-third voluntarily engaged in the POs' work (36.8%, *n* = 491). Of these, most indicated more than one volunteer activity (61.1%, *n* = 300). Most commonly, PO members volunteered to lead support groups (51.7%, *n* = 254) and to serve as advisors to other members (47%, *n* = 231) (see Supplementary Material C, Table 1 for full results on voluntary activities).

**Table 1:** Participant characteristics (*N* = 1,334).

Characteristic	*n* (%)
Age	
18–29 years	44 (3.3)
30–39 years	136 (10.2)
40–49 years	198 (14.8)
50–64 years	555 (41.6)
≥ 65 years	320 (24)
Missing^a^	81 (6.1)
Gender	
Female	896 (67.2)
Male	409 (30.7)
Non-binary or gender diverse	2 (0.1)
Missing^a^	27 (2)
Number of indicated diseases and/or disabilities^b^	
1	202 (15.1)
2–3	444 (33.3)
4–5	208 (15.6)
≥6	114 (8.5)
Not personally affected	330 (24.7)
Missing^a^	36 (2.7)
Educational attainment	
Basic secondary education certificate *(German: Hauptschulabschluss)*	64 (4.8)
Intermediate secondary education certificate *(German: Realschulabschluss)*	278 (20.8)
Entrance qualification for universities of applied sciences *(German: Fachhochschulreife)*	123 (9.2)
General university entrance qualification *(German: Abitur)*	181 (13.6)
Higher education degree^c^	616 (46.2)
No formal qualification^d^	3 (0.2)
Other qualification	29 (2.2)
Missing^a^	40 (3)
Self-rated digital skills	
Very good	449 (33.7)
Rather good	719 (53.9)
Rather poor	136 (10.2)
Very poor	15 (1.1)
Missing^a^	15 (1.1)
Membership background	
Affected individual (by disease or disability)	1,004 (75.3)
Relative of an affected individual	247 (18.5)
Other	79 (5.9)
Missing^a^	4 (0.3)
Duration of membership	
Less than 1 year	120 (9)
1–2 years	156 (11.7)
3–5 years	308 (23.1)
6–10 years	258 (19.3)
More than 10 years	492 (36.9)
Voluntary work in PO	
Yes	491 (36.8)
No	831 (62.3)
Missing^a^	12 (0.9)

^a^ Missing values include “prefer not to answer” responses and unanswered questions.

^b^ This was a filter question shown only to participants who indicated that they themselves have a disease or disability (i.e., not shown to family members or other non-affected individuals). Respondents could select all diseases and/or disabilities that applied to them. Therefore, multiple selections do not necessarily indicate multiple independent conditions but may reflect different aspects of a single underlying condition. These values should therefore be interpreted with caution. The full list of response options can be found in the questionnaire included in Supplementary Material A. Due to the filtering process, participants categorized as “Not personally affected” were technically treated as missing values for this variable. However, they are presented here as a separate category. As imputation was not appropriate — given that these participants were intentionally excluded based on their non-affected status — this variable was ultimately omitted from the logistic regression analysis to avoid an unnecessary increase in missing cases.

^c^ “Higher education degree” includes college and university degrees without differentiation between specific levels.

^d^ This category includes people who left school without a diploma or who do not yet have one.

### 3.2 Involvement in the development of digital services

#### 3.2.1 Overall findings

Of all respondents, 22.4% (*n* = 299) had been approached by their PO to get involved in the development of a digital service. Among them, 65.6% (*n* = 196) agreed to get involved, while 30.1% (*n* = 90) declined. Of those who agreed, the majority contributed to the development or design of PO websites (80.6%, *n* = 158), digital newsletters (25%, *n* = 49), and mobile apps (12.8%, *n* = 25). Additionally, 41.3% (*n* = 81) indicated having contributed to more than one digital service. Members were primarily involved in developing content (e.g., creating texts or videos; 73%, *n* = 143) and in the general planning process (e.g., as members of a working group; 66.8%, *n* = 131). Other common forms of involvement included expressing their preferences regarding the digital service (e.g., through surveys, interviews, or group discussions; 55.1%, *n* = 108) and testing a prototype (46.9%, *n* = 92). Most rated their involvement experience positively (very positive: 42.3%, *n* = 83; rather positive: 31.6%, *n* = 62). Those who declined to be involved cited a lack of time (71.1%, *n* = 64) and uncertainty about their (technical) skills (40%, *n* = 36) as the main reasons. A complete overview of the results is provided in Supplementary Material C, Tables 2–5.

#### 3.2.2 Predictors of being approached for involvement

Our multivariate logistic regression analysis indicates that POs are more likely to approach members who are volunteers or have a longer-lasting membership. Specifically, we observed that volunteers were significantly more likely to be approached than non-volunteers (OR = 5.227, 95% CI: 3.765–7.256, *p* < 0.001). Members with up to two years of membership (OR = 0.401, 95% CI: 0.240–0.671, *p* < 0.001), and those with six to ten years of membership (OR = 0.565, 95% CI: 0.363–0.879, *p* = 0.011) were less likely to be approached than members with more than ten years of membership. See Table 2 for detailed results of the multivariate logistic regression and Table 11 in Supplementary Material C for the preceding cross-tabulation analysis.

**Table 2:** Results of multivariate logistic regression analysis on being approached to get involved*.

Predictor	OR	95% CI	*p*-value
Age^a^			
18–34 years	1.346	0.666–2.721	0.408
35–49 years	1.153	0.692–1.921	0.584
50–64 years	1.364	0.911–2.041	0.131
≥ 65 years	Ref	–	–
Gender^a^			
Female	Ref	–	–
Male	1.370	0.974–1.927	0.070
Educational qualification^c^^,^^d^^,^^e^			
Basic or intermediate secondary education certificate *(German: Hauptschulabschluss or Realschulabschluss)*	1.051	0.705–1.567	0.807
Entrance qualification for universities of applied sciences *(German: Fachhochschulreife)*	1.223	0.719–2.080	0.458
General university entrance qualification *(German: Abitur)*	1.136	0.713–1.810	0.591
Higher education degree	Ref	–	–
Self-rated digital skills^f^			
Very good	Ref	–	–
Rather good	0.918	0.643–1.312	0.640
Poor	0.842	0.455–1.560	0.585
Membership background^g^			
Affected individual (by disease or disability)	Ref	–	–
Relative of an affected individual	0.956	0.638–1.431	0.826
Duration of membership^h^			
Up to 2 years	0.401	0.240–0.671	**<0.001**
3–5 years	0.701	0.468–1.051	0.085
6–10 years	0.565	0.363–0.879	**0.011**
More than 10 years	Ref	–	–
Voluntary work in PO			
Yes	5.227	3.765–7.256	**<0.001**
No	Ref	–	–

“Ref” indicates the reference category against which all other categories are compared.

Bold *p*-values indicate statistical significance at *p* < 0.05.

^a^ The age categories were adjusted due to fewer than 10 cases in the “yes” category of the outcome variable for the 18–29 group, resulting in the creation of broader age groups.

^b^ The “Non-binary or gender diverse” category was excluded from the multivariate logistic regression analysis due to the very small sample size (*n* = 2).

^c^ The category “Basic secondary education certificate” was merged with “Intermediate secondary education certificate” due to fewer than 10 cases in the “yes” category of the outcome variable for the former.

^d^ The category “No formal qualification” was excluded from the analysis due to a sample size of fewer than 10 cases, and it could not be meaningfully combined with other categories.

^e^ The category “Other qualification” was excluded from the analysis due to its heterogeneity resulting from various qualifications and its limited comparability with other education levels.

^f^ “Very poor” and “Rather poor” were combined into “Poor” due to fewer than 10 cases in the “yes” category of the outcome variable for the former.

^g^ The category “Other” comprised a heterogeneous group (e.g., healthcare professionals, former patients, volunteers, and full-time staff). Due to the group's internal diversity as well as overlap with the categories “affected individuals” and “family members”, it was excluded from further analysis.

^h^ The categories “<1 year” and “1-2 years” were merged due to fewer than 10 cases in the “yes” category of the outcome variable for the former.

* The model was significant (Omnibus Test: χ^2^ = 159.061, df = 14, *p* < 0.001), explained 21.2% of the variance (Nagelkerke R^2^ = 0.212), and showed a good fit (Hosmer-Lemeshow test: χ^2^ = 13.688, df = 8, *p* = 0.090).

#### 3.2.3 Predictors of agreeing to get involved

Our results suggest that PO members with certain characteristics, such as being volunteers, having higher self-rated digital skills, or an advanced educational background, are more likely to actually get involved in the development of digital services within their PO. Specifically, non-volunteers were significantly less likely to agree to get involved than volunteers (OR = 0.076, 95% CI: 0.032–0.181, *p* < 0.001). Members who rated their digital skills as poor (OR = 0.070, 95% CI: 0.016–0.300, *p* < 0.001), and those who rated them as rather good (OR = 0.348, 95% CI: 0.141–0.858, *p* = 0.022) were less likely to agree than members who rated their digital skills as very good. Similarly, members with a basic or intermediate secondary education certificate were less likely to agree than those with a college or university degree (OR = 0.178, 95% CI: 0.073–0.437, *p* < 0.001). See Table 3 for detailed results of the multivariate logistic regression and Table 12 in Supplementary Material C for the preliminary cross-tabulation analysis.

**Table 3:** Results of multivariate logistic regression analysis on agreeing to get involved*.

Predictor	OR	95% CI	*p*-value
Age^a^			
18–34 years	1.470	0.275–7.852	0.652
35–49 years	1.084	0.353–3.331	0.888
50–64 years	1.366	0.577–3.234	0.478
≥65 years	Ref	–	–
Gender^a^			
Female	Ref	–	–
Male	1.138	0.537–2.413	0.735
Educational attainment^a^^,^^b^			
Basic or intermediate secondary education certificate *(German: Hauptschulabschluss or Realschulabschluss)*	0.178	0.073–0.437	**<0.001**
Entrance qualification for universities of applied sciences *(German: Fachhochschulreife)*	0.437	0.140–1.368	0.155
General university entrance qualification *(German: Abitur)*	1.283	0.428–3.846	0.657
Higher education degree	Ref	–	–
Self-rated digital skills^a^			
Very good	Ref	–	–
Rather good	0.348	0.141–0.858	**0.022**
Poor	0.070	0.016–0.300	**<0.001**
Membership background^a^			
Affected individual (by disease or disability)	Ref	–	–
Relative of an affected individual	1.350	0.550–3.312	0.512
Duration of membership^a^			
Up to 2 years	3.035	0.837–11.005	0.091
3–5 years	0.538	0.211–1.372	0.194
6–10 years	1.874	0.614–5.721	0.270
More than 10 years	Ref	–	–
Voluntary work in PO			
Yes	Ref	–	–
No	0.076	0.032–0.181	**<0.001**

“Ref” indicates the reference category against which all other categories are compared.

Bold *p*-values indicate statistical significance at *p* < 0.05.

^a^ Categories were either merged or excluded following the procedure outlined in Table 2.

^b^ The category “General university entrance qualification *(German: Abitur)*” was retained despite only *n* = 8 cases in the “no” category of the outcome variable, due to its conceptual distinction from other education levels and the notably different response trend (see Supplementary Material C, Table 12).

* The model was significant (Omnibus Test: χ^2^ = 96.154, df = 14, *p* < 0.001), explained 47.2% of the variance (Nagelkerke R^2^ = 0.472), and showed a good fit (Hosmer-Lemeshow test: χ^2^ = 8.710, df = 8, *p* = 0.367).

### 3.3 Willingness to get involved

#### 3.3.1 Overall findings

**Figure 1:**
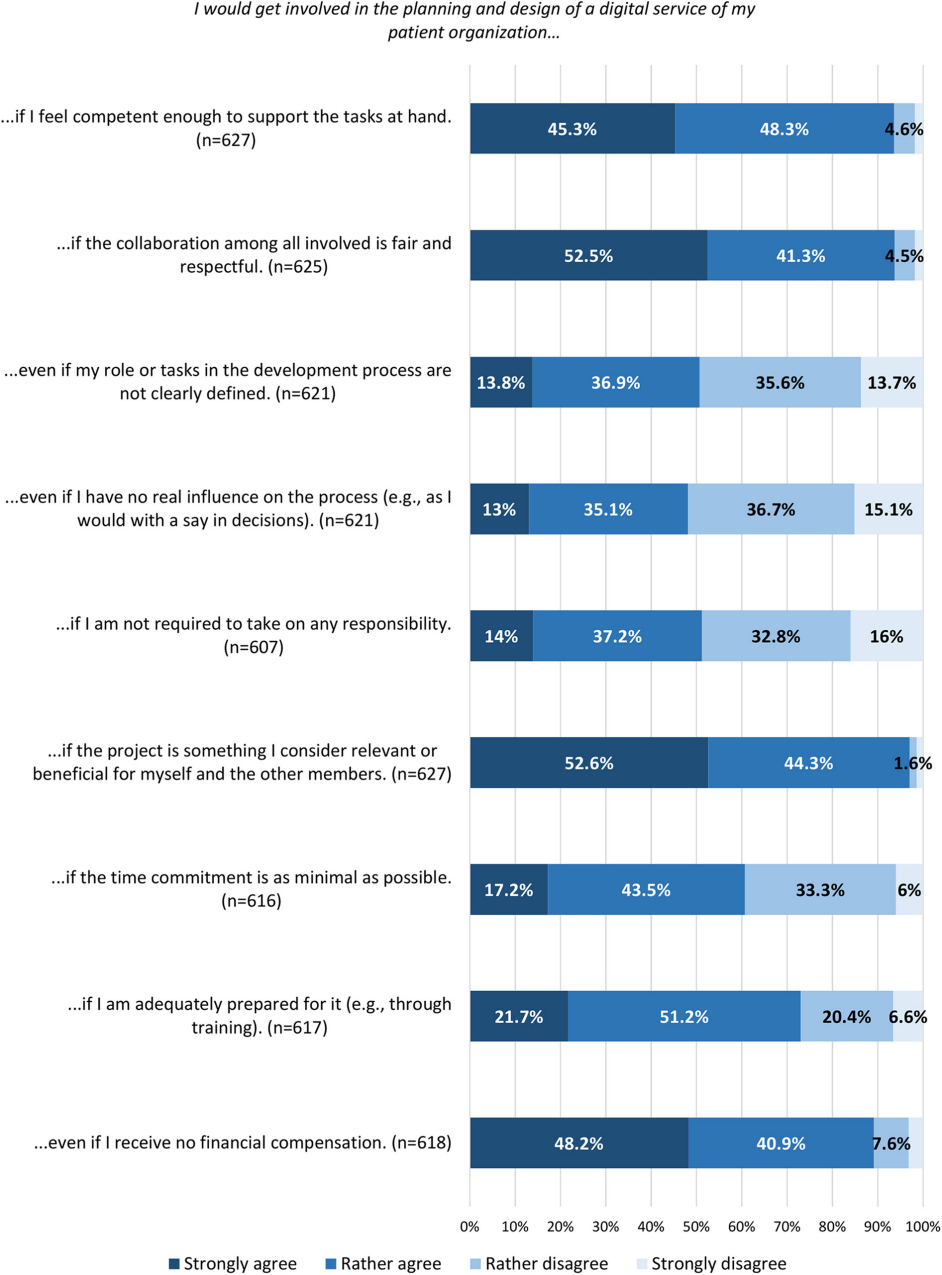
Conditions for willingness to get involved; the smallest values were removed from the bar chart when overlapping made them unreadable, but are fully documented in Supplementary Material C, Table 8.

**Figure 2:**
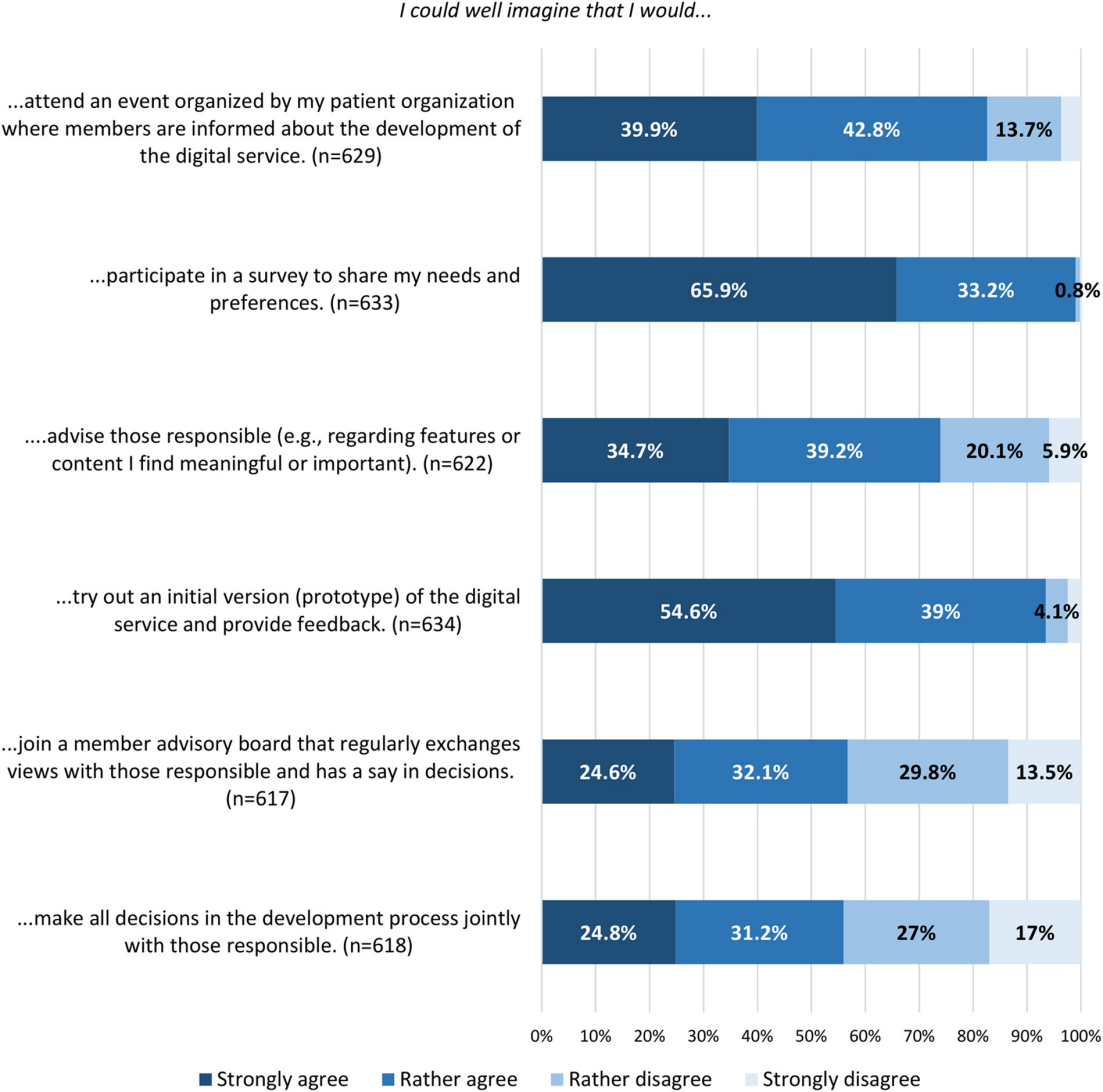
Members’ willingness to engage in different methods of involvement; the smallest values were removed from the bar chart when overlapping made them unreadable, but are fully documented in Supplementary Material C, Table 9.

The majority of respondents (81.2%, *n* = 1,083) stated that involving members in the development of new digital services within their PO is important, and 64% (*n* = 854) indicated that they would disapprove of a collaboration with an external partner on such services if members were not given the opportunity to be involved (see Supplementary Material C, Tables 6 and 7 for the full distribution of responses). Nearly half (48%, *n* = 640) expressed a general willingness to get involved, while 44% (*n* = 587) stated that they were not willing. Another 8% (*n* = 107) chose not to answer. Among those who expressed unwillingness, the main reasons were lack of time (61.8%, *n* = 336) and uncertainty about one's own (technical) skills (43.3%, *n* = 254) (see Supplementary Material C, Table 8 for detailed results). Among participants who indicated a general willingness to get involved, key conditions included the relevance of the digital PO service being designed (96.9%, *n* = 608), confidence in the ability to contribute (93.6%, *n* = 587), and equal and respectful collaboration (93.8%, *n* = 586) [see Figure 1 and Supplementary Material B (B4) for notes on variation in response counts]. Participating in surveys (99.1%, *n* = 627) and testing prototypes (93.6%, *n* = 593) received the highest levels of acceptance as involvement methods. PO members were less willing to join advisory boards (56.7%, *n* = 350) or engage in collective decision-making processes (56%, *n* = 346), both of which include some degree of decision-making authority [see Figure 2 and Supplementary Material B (B4) for notes on variation in response counts].

#### 3.3.2 Predictors of willingness to get involved

Our findings indicate that certain characteristics of PO members are associated with a greater willingness to get involved in the development of digital services, including volunteer status, younger age, male gender, higher self-rated digital skills, and higher educational attainment. Specifically, members who volunteer within their PO were significantly more likely to express willingness than non-volunteers (OR = 2.905, 95% CI: 2.163–3.901, *p* < 0.001). Compared to members aged 65 years and older, younger age groups, including those aged 30–39 years (OR = 2.022, 95% CI: 1.217–3.360, *p* = 0.007), those aged 40–49 years (OR = 1.664, 95% CI: 1.059–2.615, *p* = 0.027), and those aged 50–64 years (OR = 1.490, 95% CI: 1.052–2.110, *p* = 0.025) were more likely to express willingness. Male members were more likely to express willingness than female members (OR = 1.661, 95% CI: 1.233–2.238, *p* < 0.001). Members with poor digital skills (OR = 0.235, 95% CI: 0.135–0.407, *p* < 0.001) and members with rather good digital skills (OR = 0.720, 95% CI: 0.537–0.964, *p* = 0.027) were less likely to express willingness than members who rated their digital skills as very good. Furthermore, we found that PO members with a basic or an intermediate secondary education certificate were less likely to express willingness than those with a college or university degree (OR = 0.494, 95% CI: 0.256–0.951, *p* = 0.035; and OR = 0.614, 95% CI: 0.436–0.865, *p* = 0.005, respectively). See Table 4 for the detailed results of the multivariate logistic regression and Table 13 in Supplementary Material C for the preliminary cross-tabulation analysis.

**Table 4:** Results of multivariate logistic regression analysis on willingness to get involved*.

Predictor	OR	95% CI	*p*-value
Age			
18–29 years	1.906	0.925–3.929	0.080
30–39 years	2.022	1.217–3.360	**0.007**
40–49 years	1.664	1.059–2.615	**0.027**
50–64 years	1.490	1.052–2.110	**0.025**
≥65 years	Ref	–	–
Gender^a^			
Female	Ref	–	–
Male	1.661	1.233–2.238	**<0.001**
Educational attainment^b^			
Basic secondary education certificate *(German: Hauptschulabschluss)*	0.494	0.256–0.951	**0.035**
Intermediate secondary education certificate *(German: Realschulabschluss)*	0.614	0.436–0.865	**0.005**
Entrance qualification for universities of applied sciences *(German: Fachhochschulreife)*	1.243	0.779–1.983	0.361
General university entrance qualification *(German: Abitur)*	0.846	0.572–1.252	0.404
Higher education degree	Ref	–	–
Self-rated digital skills^a^			
Very good	Ref	–	–
Rather good	0.720	0.537–0.964	**0.027**
Poor	0.235	0.135–0.407	**<0.001**
Membership background^a^			
Affected individual (by disease or disability)	Ref	–	–
Relative of an affected individual	0.769	0.548–1.079	0.128
Duration of membership			
Less than 1 year	1.568	0.955–2.576	0.076
1–2 years	1.267	0.804–1.997	0.307
3–5 years	0.984	0.691–1.401	0.927
6–10 years	1.044	0.714–1.526	0.826
More than 10 years	Ref	–	–
Voluntary work in PO			
Yes	2.905	2.163–3.901	**<0.001**
No	Ref	–	–

“Ref” indicates the reference category against which all other categories are compared.

Bold *p*-values indicate statistical significance at *p* < 0.05.

^a^ Categories were either merged or excluded following the procedure outlined in Table 2.

^b^ As outlined in Table 2, the category “No formal qualification” was excluded from the analysis. However, the categories “Basic secondary education certificate” and “Intermediate secondary education certificate” were not merged, as neither had fewer than 10 cases in any category of the outcome variable.

* The logistic regression model was significant (Omnibus Test: χ^2^ = 142.340, df = 17, *p* < 0.001), explained 17.2% of the variance (Nagelkerke R^2^ = 0.172), and showed a good fit (Hosmer-Lemeshow test: χ^2^ = 5.696, df = 8, *p* = 0.681).

We further examined willingness to engage in different involvement formats using cross-tabulations to analyze differences across the independent variables included in the logistic regression models (see Supplementary Material C, Table 14–19 for details). Self-rated digital skills were significantly associated with greater willingness to participate in surveys, advise decision-makers, and test prototypes, while no significant association was found for formats involving decision-making authority. Higher educational attainment was significantly linked to greater willingness to advise decision-makers, test prototypes, and join an advisory board with a say in decisions. A significantly higher proportion of volunteers than non-volunteers expressed willingness to advise decision-makers, join advisory boards, and take part in joint decision-making. Hence, digital skills, education, and volunteer status may play a role in members' willingness to engage in certain involvement formats.

## 4 Discussion

In this nationwide survey, we explored PO members' perspectives on involvement in the development of digital PO services. Our findings provide valuable insights into members' willingness to engage, actual involvement, as well as predictive factors.

### 4.1 Key findings

Most PO members considered it important that members are involved in the development of digital PO services. This view was echoed in participants' disapproval of external collaborations that excluded member involvement — suggesting that involvement is not only valued, but in some cases seen as a prerequisite. Nearly half of the respondents expressed a willingness to personally contribute to development efforts within their PO. In our sample, willingness to engage in forms of involvement without direct decision-making power, such as surveys or prototype testing, was highest. Additionally, participants indicated that perceiving a digital service as beneficial for both themselves and others, as well as feeling competent enough to contribute, are key prerequisites for involvement. While many members expressed a willingness to engage, fewer had been approached by their POs. Among those approached, the majority agreed to get involved, demonstrating considerable willingness when asked. Members were primarily involved in developing or designing PO websites, mainly by contributing content (e.g., videos or texts) and participating in general planning processes. Volunteering within the PO and high self-rated digital skills were significantly associated with greater willingness and a higher likelihood of agreeing to be involved. Additionally, members who volunteered were more likely to be approached by their PO. Uncertainty about one's own skills and limited time resources were cited as the main reasons for unwillingness or refusal to get involved.

### 4.2 Contextualization of findings

#### 4.2.1 Key predictors of willingness and actual involvement

As volunteer activity and self-rated digital skills emerged as significant predictors of different aspects of involvement, this section explores potential mechanisms underlying these relationships, drawing on insights from previous research. Other independent variables, such as age, gender, and educational attainment, also showed significant associations in some of our analyses, but were less consistent, either being significantly associated with only one of the three involvement outcomes or demonstrating significance for only certain categories.

##### 4.2.1.1 Volunteer activity

The significant associations between volunteer activity and all three involvement outcomes could be driven by several factors. Research exploring differences in the *Big Five* personality traits suggests that volunteers tend to exhibit higher levels of these traits than non-volunteers (24–26). These findings may help explain why those already actively volunteering within their PO show greater willingness. For instance, the altruism and community-focused attitude underlying *Agreeableness* (27) may increase volunteers' motivation to contribute to initiatives that benefit others, such as digital services designed to support PO members. Similarly, the open-mindedness and curiosity associated with *Openness to Experience* (27) might encourage volunteers to engage in potentially innovative or creative projects, such as co-developing a new digital PO service. The sense of duty, responsibility, and reliability linked to *Conscientiousness* (27) may make volunteers more willing to take on additional responsibilities. The sociability inherent in *Extraversion* (27) could act as a motivator, as collaborative processes provide opportunities for interaction and teamwork. Research has also shown that volunteers tend to exhibit higher levels of self-efficacy (28, 29). According to Bandura (30) self-efficacy refers to an individual's belief in their ability to successfully handle tasks and situations. It can therefore be assumed that PO members who engage in voluntary work also tend to have higher self-efficacy. This, in turn, may increase their willingness to also contribute to the development of new digital services, as they likely feel more confident in their ability to do so. As self-efficacy and the *Big Five* personality traits were not directly measured in this study, our considerations remain speculative. Future research could explore the characteristics that distinguish PO members who engage in formal volunteering from other members, potentially allowing for more concrete conclusions regarding the aspect of willingness to get involved.

One possible reason why volunteers are more likely to be approached by their POs for involvement could be their existing active roles, such as leading support groups or serving on committees. This may increase their visibility within their POs and, combined with their demonstrated commitment and familiarity with certain organizational processes, may lead decision-makers to view them as more accessible, reliable, and willing candidates for additional involvement. Moreover, our earlier qualitative interview study showed that volunteers were frequently involved in ongoing digital tasks, such as maintaining their PO's website or managing its social media channels (12). This kind of practical experience may strengthen decision-makers' confidence in their competence and reliability-especially when considering them for involvement in the development and design of new digital services. Another possible explanation is that mobilizing members for volunteer work is generally difficult for many German POs (9). This could prompt decision-makers to further rely on already engaged volunteers, perceiving them as accessible and dependable. However, the same research also highlights that these volunteers are often overburdened with existing responsibilities. Assigning them additional tasks would increase their workload and heighten the risk of burnout or disengagement (31).

To avoid overburdening volunteers, POs could explore strategies to involve non-volunteers more effectively. Given the challenges POs face in recruiting members for active roles and the fact that time constraints emerged as a common barrier to involvement in our study, offering flexible engagement options could be beneficial. One such approach is *microvolunteering*, which involves small, manageable tasks requiring minimal time commitment (32), which could appeal to those with limited availability. Furthermore, our findings suggest that respondents were more willing to engage in lower-commitment formats and hence, providing different involvement options could increase accessibility for diverse member groups. Virtual engagement opportunities could also help reduce barriers, such as the need to travel (33). Overall, diversifying involvement formats — for instance, by offering both online and in-person options — may help better accommodate members' needs and preferences, enabling POs to reach individuals who might otherwise hesitate to get involved.

##### 4.2.1.2 Digital skills

Members with higher self-rated digital skills were significantly more likely to express willingness and agree to get involved when approached by their PO. In addition, feeling competent enough to contribute meaningfully was seen as a key prerequisite for engagement, whereas a perceived lack of ability was often cited as a reason for disinterest or refusal. This could again be related to Bandura's (30) concept of self-efficacy. In our context, members with higher perceived digital competence may feel more confident in their ability to make meaningful contributions. While our assessment focused on general perceptions of digital competence rather than task-specific self-efficacy, fostering such confidence among hesitant members through initiatives aimed at improving digital and other required skills may encourage broader member involvement. This aligns with patient and public involvement (PPI) research, which emphasizes the importance of building necessary competencies in advance if they are lacking among those expected to be involved (34, 35). The German PO *Deutsche Rheuma-Liga* demonstrates this by proactively training its members in advance of research projects (36). However, implementing such measures will likely require substantial financial and human resources, which could be challenging for many German POs already facing resource constraints (1, 9).

#### 4.2.2 Discrepancy between willingness and actual involvement

Our findings also reveal a notable discrepancy between members' willingness to engage and their actual involvement within our sample. This observation aligns with indications of such a gap identified in our earlier qualitative interview study (12). These findings suggest untapped involvement potential within German POs. While our study did not directly investigate the reasons for this disparity, previous research provides insights that may help explain it.

##### 4.2.2.1 Resource constraints

One possible factor contributing to this discrepancy could be resource constraints, as limited financial and personnel resources remain a fundamental challenge for many German POs (1, 9). Implementing involvement processes often requires substantial investments of time, financial, and human resources. This is illustrated, for example, by research on PPI, which highlights the organizational capacity required to manage such efforts (18, 19, 37). While these findings are not specific to POs, they exemplify the general resource-intensive nature of involvement processes, which may also apply in the context of POs.

##### 4.2.2.2 Membership demographics

Previous research has shown that many POs face an aging membership (9). In our sample, younger members were significantly more likely to express willingness to get involved than those aged 65 and older. Hence, POs with a large proportion of older members may experience lower overall involvement readiness. As a result, fewer involvement opportunities may be initiated, possibly due to a perceived lack of demand, which in turn could contribute to the observed lower actual involvement. Our findings suggest that lower self-rated digital skills may play a role in the lower willingness observed among older members, as we identified a declining trend in digital skills with age (see Supplementary Material C, Table 20). This aligns with research linking older age to lower digital health literacy (38, 39). As previously discussed, lower self-rated digital skills were significantly associated with a lower likelihood of expressing willingness to get involved. Further research is needed to better understand the interplay between an aging membership, digital skills, and involvement dynamics in digital initiatives within POs.

##### 4.2.2.3 Perspectives of decision-makers

Nickel et al. (11) found that older board members of POs were, in some cases, perceived as a barrier to digitalization, largely due to concerns about being overwhelmed by new technologies. This restrictive stance may result in limited digitalization efforts and, consequently, fewer opportunities for member involvement. Such dynamics may contribute to the relatively low engagement rates in our sample. Another contributing factor could be a lack of experience in implementing involvement processes or limited awareness among decision-makers of the benefits of involving members. In line with this, research on PPI suggests that when researchers lack such competencies, they need to be developed before successful PPI processes can take place (40).

##### 4.2.2.4 Digitalization levels

Another aspect is the varying levels of digitalization among POs, many of which are still in the early stages (11). Here, efforts may focus primarily on building basic digital infrastructure, leaving limited opportunities for member involvement, as it may not yet be feasible or prioritized in less digitized POs. However, this remains speculative and highlights the need for further research.

### 4.3 Strengths and limitations

A major strength of our study is its large sample size of 1,334 participants and nationwide data collection, which provides broad insight into the perspectives and experiences of members of German POs regarding their involvement in the development of digital services. This is particularly noteworthy given the indirect recruitment approach, which relied on POs to forward the study invitation to their members and limited our direct access and control over dissemination. As discussed earlier, many POs face challenges in engaging their members, which further underscores the strength of this response. The sample size also aligns with that reported in a large-scale survey of German self-help groups, which are often part of or closely affiliated with POs (41).

However, certain aspects should be considered when interpreting the scope of our findings. One such aspect is the demographic composition of our sample, which was predominantly female, older, and highly educated. While no representative data are available on the overall membership profile of POs in Germany, the aforementioned survey of self-help group members — many of which are organizationally linked to POs — revealed comparable patterns. This lends support to the assumption that our sample captures common demographic features of individuals engaged in the broader self-help and patient organization landscape (41). Nevertheless, self-selection bias cannot be ruled out — for instance, if individuals from these demographic groups are generally more willing to participate in research. Another potential source of this bias may relate to the topic itself, as members with a strong interest in digitalization or digital tools may have been more inclined to take part in the study. Another limitation concerns socially desirable responses, which may have affected the accuracy of self-reported data. Additionally, the online survey format may have excluded members without adequate digital access or competencies. Unequal subgroup sizes, such as the small number of participants with very poor digital skills or from younger age groups, could have impacted the statistical reliability for these groups. We addressed this issue by merging categories with small cell counts in our logistic regression analyses to ensure sufficient sample sizes, thereby enhancing the robustness of our models. Our logistic regression models for the dependent variables “general willingness to get involved” and “being approached by the PO” explained 17.2% and 21.2% of the variance, respectively. Although the models identified robust predictors, this relatively low explained variance suggests that additional unmeasured factors likely affect the likelihood of PO members being approached or their willingness to get involved. Lastly, involvement approaches to digital initiatives within POs may have been implemented without all members' awareness. However, we believe that our sample size has allowed us to identify genuine trends.

## 5 Conclusion

This study demonstrates a considerable willingness among PO members to be involved in the development of digital services, but relatively few opportunities for actual involvement. Key factors influencing both willingness and actual involvement include volunteer activity and self-rated digital skills. To bridge the gap between willingness and practice, and thereby unlock untapped involvement potential, PO leaders may consider strategies to expand involvement beyond their volunteer base, address digital literacy barriers, and tailor approaches to accommodate members’ time constraints and preferred levels of responsibility. Flexible involvement options, such as *microvolunteering* and targeted outreach to previously underrepresented groups, seem warranted to increase engagement and avoid overburdening volunteers. This approach may allow POs to better leverage the perspectives of all members. Incorporating these perspectives into digital services, as well as broader digital transformation efforts within POs, could enhance the relevance and effectiveness of these initiatives. Future research should explore how organizational and structural factors, such as resource allocation and organizational culture, affect the feasibility of involvement processes within POs.

## Data Availability

The dataset analyzed in this article is part of a broader survey and is not readily available in a form suitable for public sharing. Requests for access will be reviewed on a case-by-case basis in accordance with data protection regulations and institutional policies. Please direct inquiries to Simon Wallraf (wallraf.simon@mh-hannover.de).
