# Supplementary material for: Between willingness and practice: a nationwide survey of 1,334 German patient organization members on user involvement in digital service development
Source: Front Digit Health. 2025 Jun 6;7:1591981. doi: 10.3389/fdgth.2025.1591981 (PMC12179215; doi:10.3389/fdgth.2025.1591981)
Supplement: Supplementary file 1 [file Datasheet1.pdf]

Supplementary Material A

Digitalization in Patient Organizations: Survey Questionnaire

Section ND: Use of Digital Technologies and Services

The healthcare sector is undergoing a digital transformation. Digital technologies and services are creating new and increasingly diverse opportunities, for example, to access information, utilize healthcare services, connect with others, or store personal health data. Therefore, we are first interested in understanding whether and which digital technologies and services you use for your health-related concerns and questions. Below, we have listed some digital options.

[ND02] Multiple Choice

"Which digital technologies and/or services do you use in your daily life for health-related concerns and questions?"

ND02_01 Internet (e.g., websites, search engines)

ND02_02 Emails

ND02_03 Messaging services (e.g., WhatsApp, Signal)

ND02_04 Video conferencing services (e.g., Zoom, Skype)

ND02_05 Social media (e.g., Facebook, Twitter, Instagram, TikTok)

ND02_06 Video and/or music streaming services (e.g., YouTube, Netflix, Spotify)

ND02_07 Podcast streaming services (e.g., Spotify, Apple Podcasts)

ND02_08 Video consultations

ND02_09 Telemonitoring (e.g., via app or wearable device)

ND02_10 Online pharmacies

ND02_11 Health and fitness apps (e.g., *DiGAs*)

ND02_12 Electronic health record

ND02_13 Electronic medication plan

ND02_14 Electronic vaccination record

ND02_15 Electronic maternity record

ND02_16 Electronic prescription

Answer coding:

1 = Not selected

2 = Selected

ND02_17a Other digital technologies/services

Open text input

Alternative option

-1 = I do not use any digital technologies/services for my health-related concerns or questions.

[ND03] Multiple Choice| **Filter Question: ND03 is only displayed if the respondent selected "no digital technologies/services" under ND02.**

"Why do you not use digital technologies/services for your health-related concerns and questions?"

ND03_01 …I do not know which digital technologies/services are suitable for me.

ND03_02 …I have concerns about the security of my data.

ND03_03 …I prefer to consult with healthcare professionals in person.

ND03_04 Other reasons

Answer coding:

1 = Not selected

2 = Selected

ND03_04a Other reasons

Open text input

[ND04] Single Choice

"How would you generally rate your personal ability to use and navigate digital technologies and/or services?"

Answer coding:

1 = Very good

2 = Rather good

3 = Rather poor

4 = Very poor

-1 = No response

-9 = Not answered

[ND05] Scale Question

"We would now like to understand whether certain factors generally make it more difficult for you to use digital technologies and/or services."

I find it difficult to use digital technologies/services when...

ND05_01 …they are not accessible to me (e.g., lack of options for subtitles, text magnification, or voice control).

ND05_02 …they are structured in a complicated way, making it difficult for me to find what I am looking for.

ND05_03 …they contain a lot of information, which makes me feel overwhelmed.

ND05_04 …I do not fully understand what happens to my personal data.

ND05_05 …texts and information are written in technical jargon.

ND05_06 …they require a fast internet connection.

ND05_07 …they require a smartphone, and I need to be familiar with its functions.

ND05_08 …when I need to have good computer skills.

Answer coding:

1 = Strongly disagree

2 = Rather disagree

3 = Rather agree

4 = Strongly agree

-1 = No response

-9 = Not answered

[ND06] Open Text Input

Other difficulties (regarding ND05)

ND06_01 Other difficulties, namely:

Open text input

[ND07] Open Text Input

“Is there anything else you would like to share with us regarding the use of digital technologies and services?”

Open text input

Section MI: Membership in Patient Organizations

In the following, we would like to gather information about your membership in your patient organization.

[MI02] Single Choice

“Are you a member of a patient organization?”

1 = Yes
2 = No
-9 = Not answered

[MI03] Single Choice

“Which of the following best describes the reason for your membership?”

Answer coding:

1 = Person affected (by disease/disability)

2 = Family member of an affected person

3 = Other, namely:

-1 = No response

-9 = Not answered

MI03_03 Other, namely

Open text input

[MI04] Single Choice

“How long have you been a member of your patient organization?”

Answer coding:

1 = Less than 1 year

2 = 1 to 2 years

3 = 3 to 5 years

4 = 6 to 10 years

5 = More than 10 years

-9 = Not answered

[MI05] Single Choice

“In addition to your membership, are you currently engaged in voluntary work within your patient organization?“

Answer coding:

1 = Yes

2 = No

-1 = No response

-9 = Not answered

[MI06] Multiple Choice | **Filter Question: MI06 is only displayed if MI05 was answered with “yes.”**

“Please check all voluntary roles you currently take on in your patient organization.”

MI06_01 Leader or spokesperson of a support group

MI06_02 Member of a regional working group, committee or forum

MI06_03 Member of a national working group, committee or forum

MI06_04 Advisor for other members and affected individuals

MI06_05 Research partner (e.g., involvement in studies)

MI06_06 Navigator/Guide

MI06_07 Consultant

MI06_08 Spokesperson

MI06_09 Treasurer

MI06_10 Other activities

Answer coding:

1 = Not selected

2 = Selected

MI06_10a Other activities, namely:

Open text input

Section DO: Digital Services in Patient Organizations

In the following, we would like to ask you some questions about the digital services provided by your patient organization.

[DO02] Single Choice (Per Item)

"Does your organization offer the following digital services?"

DO02_01 Website

DO02_02 Email communication

DO02_03 Digital newsletter

DO02_04 Online counseling (e.g., via Zoom, BigBlueButton)

DO02_05 Online support groups

DO02_06 Online training / webinars (e.g., via Zoom, BigBlueButton)

DO02_07 Online discussion forum

DO02_08 Social media services (e.g., Facebook, Instagram, Twitter)

DO02_09 Digital patient registry

DO02_10 Communication via messaging services (e.g., WhatsApp or Signal)

DO02_11 Online surveys or polls (e.g., via Doodle)

DO02_12 Own mobile app

DO02_13 YouTube channel

DO02_14 Video content (e.g., video tutorials or event recordings)

DO02_15 Podcast / audio content

Answer coding:

1 = Yes

2 = No

-1 = Don't know

-9 = Not answered

[DO03] Single Choice + Open Text Input

Other digital services (regarding DO02)

Answer coding:

1 = Other, namely:

+ Open text input

-9 = Not answered

[DO04] Scale Question | **Filter Question: Only the digital services selected under DO02 are displayed.**

“How often do you use these digital services provided by your patient organization?”

DO04_01 Website

DO04_02 Email communication

DO04_03 Digital newsletter

DO04_04 Online counseling (e.g., via Zoom, BigBlueButton)

DO04_05 Online support groups

DO04_06 Online training / webinars (e.g., via Zoom, BigBlueButton)

DO04_07 Online discussion forum

DO04_08 Social media services (e.g., Facebook, Instagram, Twitter)

DO04_09 Digital patient registry

DO04_10 Communication via messaging services (e.g., WhatsApp or Signal)

DO04_11 Online surveys or polls (e.g., via Doodle)

DO04_12 Own mobile app

DO04_13 YouTube channel

DO04_14 Video content (e.g., video tutorials or event recordings)

DO04_15 Podcast / audio content

Answer coding:

1 = Never

2 = Less than once per month

3 = Once per month

4 = About every two weeks

5 = Once per week

6 = Several times per week

7 = Daily

-9 = Not answered

[DO05] Open Text Input

“How often do you use the digital service you specified under 'Other'?” (regarding DO04)

Open text input

[DO06] Single Choice + Open Text Input

“How satisfied are you with the range of digital services offered by your patient organization?”

Answer coding:

1 = Very satisfied, because:

+ Open text input

2 = Rather satisfied, because:

+ Open text input

3 = Rather dissatisfied, because:

+ Open text input

4 = Very dissatisfied, because:

+Open text input

-1 = No response
-9 = Not answered

[DO07] Open Text Input

“What additional digital services would you like your patient organization to offer?“

Open text input

Section PA: Member Involvement in the Planning and Design of Digital Services

Please imagine the following situation:

Your organization plans to redesign its website or develop an app for its members. These new services would allow you to participate in surveys, read current information from your organization, access information about diseases or disabilities, upload your own data, and communicate more easily with other members. To design these services (e.g., deciding which functions to include, how content should be presented, etc.), your organization invites you and other members to get involved in the development process.

Please answer the following questions regarding this situation.

[PA02] Single Choice

“Would you generally be interested in getting involved in the planning and design of a digital service of your patient organization?“

Answer coding:

1 = Yes

2 = No

-1 = No response

-9 = Not answered

[PA03] Scale Question | **Filter Question: PA03, PA04, and PA05 are only displayed if PA02 was answered with “yes.”**

“The following statements refer to different ways of getting involved in the planning and design of a digital service.“

I could well imagine that I would…

PA03_01 ...attend an event organized by my patient organization where members are informed about the development of the digital service.

PA03_02 ...participate in a survey to share my needs and preferences.

PA03_03 ...advise those responsible (e.g., regarding features or content I find meaningful or important).

PA03_04 ...try out an initial version (prototype) of the digital service and provide feedback.

PA03_05 ...join a member advisory board that regularly exchanges views with those responsible and has a say in decisions.

PA03_06 ...make all decisions in the development process jointly with those responsible.

Answer coding:

1 = Strongly disagree

2 = Strongly disagree

3 = Rather agree

4 = Strongly agree

-1 = No response

-9 = Not answered

[PA04] Scale Question

"The following statements refer to the conditions under which you would be willing to get involved in the planning and design of a digital service for your organization."

I would get involved in the planning and design of a digital service of my patient organization…

PA04_01 ...if I feel competent enough to support the tasks at hand.

PA04_02 ...if the collaboration among all involved is fair and respectful.

PA04_03 ...even if my role or tasks in the development process are not clearly defined.

PA04_04 ...even if I have no real influence on the process (e.g., as I would with a say in decisions).

PA04_05 ...if I am not required to take on any responsibility.

PA04_06 ...if the project is something I consider relevant or beneficial for myself and the other members.

PA04_07 ...if the time commitment is as minimal as possible.

PA04_08 ...if I am adequately prepared for it (e.g., through training).

PA04_09 ...even if I receive no financial compensation.

Answer coding:

1 = Strongly disagree

2 = Strongly disagree

3 = Rather agree

4 = Strongly agree

-1 = No response

-9 = Not answered

[PA05] Open Text Input

Other conditions (regarding PA04)

Open text input

[PA06] Multiple Choice | **Filter Question: PA06 is only displayed if PA02 was answered with "no."**

"Why would you not be interested in getting involved in the planning and design of digital services in your patient organization?"

I would not be interested in getting involved because I...

PA06_01 ...do not have the time.

PA06_02 ...do not see any personal benefit in getting involved.

PA06_03 ...am not sure if my (technical) skills are sufficient.

PA06_04 ...have had negative experiences with a similar project.

PA06_05 ...am generally not interested in digital services.

PA06_06 Other reasons

Answer coding:

1 = Not selected

2 = Selected

PA06_06a Other reasons

Open text input

[PA07] Single Choice

"Has your organization ever asked you to get involved in the planning and design of digital services?"

Answer coding:

1 = Yes

2 = No

-1 = No response

-9 = Not answered

[PA08] Single Choice | **Filter Question: PA08 is only displayed if PA07 was answered with "yes."**

"Did you agree to get involved?"

Answer coding:

1 = Yes

2 = No

-1 = No response

-9 = Not answered

[PA09] Multiple Choice | **Filter Question: PA09, PA10, and PA11 are only displayed if PA08 was answered with "yes."**

"Which digital service was developed?"

PA09_01 Website

PA09_02 App

PA09_03 Digital patient registry

PA09_04 Digital newsletter

PA09_05 Other, namely:

Answer coding:

1 = Not selected

2 = Selected

PA09_05a Other, namely:

Open text input

[PA10] Multiple Choice

"**Which of the following activities were you involved in?"**

PA10_01 Expressing preferences (e.g., via a survey, interview, group discussion).

PA10_02 Planning of the digital service (e.g. as a member of a working group).

PA10_03 Developing content (texts, videos) for the digital service.

PA10_04 Designing a prototype.

PA10_05 Testing and evaluating a prototype.

PA10_06 Recommending the digital service to others.

PA10_07 Training other members on how to use the digital service.

PA10_08 Other activities, namely:

Answer coding:

1 = Not selected

2 = Selected

PA10_08a Other activities, namely:

Open text input

[PA11] Single Choice + Open Text Input

"How would you rate your overall experience of getting involved in these activities?"

Answer coding:

1 = Very positive, because:

+ Open text input

2 = Rather positive, because:

+ Open text input

3 = Rather negative, because:

+ Open text input

4 = Very negative, because:

+ Open text input

-1 = No response
-9 = Not answered

[PA12] Multiple Choice | **Filter Question: PA12 is only displayed if PA08 was answered with "no."**

"Why did you decline the request from your patient organization to get involved?"

I declined this request because...

PA12_01 ...did not have the time.

PA12_02 ...did not see any personal benefit in the digital service.

PA12_03 ...was not sure if my (technical) skills were sufficient.

PA12_04 ...was not sufficiently informed.

PA12_05 …had a negative experience with a similar project.

PA12_06 Other reasons

Answer coding:

1 = Not selected

2 = Selected

PA12_06a Other reasons

Open text input

[PA13] Texteingabe offen

"Möchten Sie uns noch etwas zum Thema „Beteiligung von Mitgliedern an der Planung und Gestaltung von digitalen Angeboten" mitteilen?"

Offene Texteingabe

Section KO: Collaborating with External Partners

Please imagine the following situation:

Together with some technically skilled members, your patient organization has developed a digital service that allows members to independently document symptoms and medication side effects. Members have full control over their data. Many members use this service and find it helpful. However, the digital service is not yet fully developed. he board, therefore, proposes to professionally advance the digital service. Since the necessary resources (e.g., financial means or technical expertise) are lacking, your organization plans to collaborate with an external partner. The digital service will then also be made available to users outside your organization.

Please answer the following questions regarding this situation.

[KO02] Scale Question

“For the (further) development of a digital service, various collaborators may be considered. How would you evaluate a collaboration between your patient organization and each of the following?“

Collaboration with…

KO02_01 ...another patient organization.

KO02_02 ...a technology company / software developer.

KO02_03 ...a public authority (e.g., health department).

KO02_04 ...a research institution (e.g., university).

KO02_05 ...a religious organization.

KO02_06 ...a hospital group / hospital network.

KO02_07 ...a health insurance provider.

KO02_08 ...a medical technology manufacturer.

KO02_09 ...a pharmaceutical company.

Answer coding:

1 = Very negative

2 = Rather negative

3 = Rather positive

4 = Very positive

-1 = No response

-9 = Not answered

[KO03] Scale Question

“What could make you disapprove of a collaboration between your patient organization and an external collaborator?”

I would disapprove of a collaboration if...

KO03_01 ...I cannot transparently understand the intentions and interests of the collaborator.

KO03_02 ...I have heard negative things about the collaborator (e.g., from the media or my social network).

KO03_03 …the collaborator does not align with my personal values.

KO03_04 ...my patient organization does not receive financial contributions from the collaborator.

KO03_05 ...my patient organization does not retain primary responsibility for the development process of the digital service.

KO03_06 ...there is no opportunity for me or other members to be involved in the development process.

KO03_07 ...the collaborator gains ownership rights to the digital service (e.g., property or usage rights).

Answer coding:

1 = Strongly disagree

2 = Strongly disagree

3 = Rather agree

4 = Strongly agree

-1 = No response

-9 = Not answered

[KO04] Open Text Input

Other reasons (regarding KO03)

Open text input

[KO05] Scale Question

“How would you generally view the further development of digital services in your patient organization?“

I would consider it positive if my patient organization…

KO05_01 ...continues to develop its existing digital services, for example, by adding new functions.

KO05_02 ...involves external partners in such further development processes.

KO05_03 ...makes the enhanced digital services available exclusively to its own members.

Answer coding:

1 = Strongly disagree

2 = Strongly disagree

3 = Rather agree

4 = Strongly agree

-1 = No response

-9 = Not answered

[KO06] Open Text Input

“Would you like to share any additional thoughts on the topic of 'Collaboration with Cooperation Partners'?“

Open text input

Section DD: Digital Data Donation

Please imagine the following situation:

To proactively advance research in its area of focus (disease/disability), your organization aims to digitally collect the health data of all its members (e.g., in its own digital patient registry). These data may include information on disease progression as well as medical or care services received. The data will be stored in an anonymized format by your organization and then made available for

research purposes. Your organization is asking you to voluntarily “donate” your data so that your information can also contribute to research projects.

Please answer the following questions regarding this situation.

[DD02] Scale Question

“To what extent would you be willing to digitally provide your health data to your patient organization?“

I would be willing to do so…

DD02_01 ...because it could help improve treatment or care options for my disease or disability.

DD02_02 ...because I can contribute to improving the treatment or care of other affected individuals.

DD02_03 ...because other members and those in charge of my patient organization expect it from me.

DD02_04 ...even if I am unsure about how my data is protected.

DD02_05 ...even if I have to send my data via a messaging service (e.g., WhatsApp).

DD02_06 ...if I receive financial compensation for it.

DD02_07 ...if research ideas from members of my patient organization are also considered.

DD02_08 ...even if my data can be used for research by commercial entities (e.g., pharmaceutical companies).

DD02_09 ...if I can decide each time which research projects my data will be used for.

DD02_10 ...to strengthen my patient organization’s influence on research.

Answer coding:

1 = Strongly disagree

2 = Strongly disagree

3 = Rather agree

4 = Strongly agree

-1 = No response

-9 = Not answered

[DD03] Open Text Input

“Would you like to share anything else on the topic of 'Digital Data Donation'?“

Open text input

Section ZE: Final Reflections on the Importance of Digital Transformation in Patient Organizations

Digitalization in patient organizations takes place at different levels, and you have already provided us with valuable insights regarding your perspectives on *implementation, involvement*, and *cooperation*.

[ZE02] Scale Question

"Finally, we would like to ask how you assess the following aspects of digitalization in your patient organization and its future development."

How important do you think it is that your patient organization…

ZE02_01 ...further develops its digital infrastructure and expands its digital services?

ZE02_02 ...involves members in the design of new digital services?

ZE02_03 ...ensures that all members, regardless of their abilities, can use digital services?

ZE02_04 ...supports members in using digital services (e.g., through training or consultation)?

ZE02_05 ...maintains non-digital ("analog") alternatives alongside digital services (e.g., in paper format or in-person)?

ZE02_06 ...does not lose sight of the importance of personal connections for emotional bonding among members?

ZE02_07 ...drives forward research projects by collecting digital health data from members (e.g., via a patient registry)?

ZE02_08 ...advocates for greater digitalization in the healthcare system (e.g., through political advocacy)?

ZE02_09 ...is actively involved in the development of new digital health services (e.g., as a collaborating partner)?

Answer coding:

1 = Not important at all

2 = Rather unimportant

3 = Rather important

4 = Very important

-1 = No response

-9 = Not answered

[ZE03] Open Text Input

"Finally, do you have any additional remarks or comments on the digital transformation of your patient organization?"

Open text input

Section SD: Demographic Information

Please provide us with some final information about yourself.

[SD02] Selection Question

"What is your gender?"

SD02 Gender

Answer coding:

1 = Female

2 = Male

3 = Non-binary or gender diverse

-1 = Prefer not to say

-9 = Not answered

[SD03] Selection + Open Text Input

"How old are you?"

SD03 Age

Answer coding:

1 = I am:

+ Open text input

-1 = Prefer not to say

-9 = Not answered

[SD04] Selection Question

"What is your highest level of formal education?"

SD04 Educational attainment

Answer coding:

1 = Degree from a university or a university of applied sciences

2 = General university entrance qualification (Abitur)

3 = Entrance qualification for universities of applied sciences (Fachhochschulreife)

4 = Intermediate secondary education certificate *(ISCED-2, Realschulabschluss)*

5 = Basic secondary education certificate *(ISCED-2, Hauptschulabschluss)*

6 = Left school without a certificate

7 = No school certificate yet

8 = Other qualification:

+ Open text input

-1 = Prefer not to say

-9 = Not answered

[SD05] Multiple Choice + Open Text Input | **Filter question: SD05 is only displayed if MI03 = 1 (i.e. respondent is personally affected by an illness/disability).**

“What (chronic) illness(es) and/or disability(ies) do you have?”

SD05_01 Musculoskeletal disorder

SD05_02 Mental illness

SD05_03 Neurological disorder

SD05_04 Cardiovascular disease

SD05_05 Respiratory disease

SD05_06 Digestive system disorder

SD05_07 Type 2 diabetes mellitus

SD05_08 Rheumatic disease

SD05_09 Cancer

SD05_10 Krebs

SD05_11 Other illness

SD05_12 Loss or partial loss of limbs

SD05_13 Impairment of limb function

SD05_14 Spinal or trunk function impairment, chest deformity

SD05_15 Blindness or visual impairment

SD05_16 Speech or hearing impairment, deafness, balance disorders

SD05_17 Loss of one or both breasts, disfigurement, etc.

SD05_18 Impairment of internal organ function

SD05_19 Paralysis, cerebral disorders, intellectual disabilities, addiction disorders

SD05_20 Other disability

Answer coding:

1 = Not selected

2 = Selected

-1 = I have neither a (chronic) illness nor a disability.

-2 = Prefer not to say

SD05_11a Other illness

SD05_20a Other disability

Open text input

[SD06] Selection + Open Text Input | **Filter question: SD06 is only displayed if MI03 = 2 (i.e., respondent is a relative of an affected person).**

“You previously indicated that you are a relative of a person with an illness/disability. We would now like to know your relationship to that person.”

I am:

Answer coding:

1 = Mother / Father

2 = Sister / Brother

3 = Spouse / Life partner

4 = Daughter / Son

5 = Grandmother / Grandfather

6 = Granddaughter / Grandson

7 = Aunt / Uncle

8 = Cousin

9 = Sister-in-law / Brother-in-law

10 = Niece / Nephew

11 = Mother-in-law / Father-in-law

12 = Stepmother / Stepfather

13 = Stepsister / Stepbrother

14 = Other:

+ Open text input

-1 = Prefer not to say

-9 = Not answered

[SD07] Selection + Open Text Input

“Finally, you have the option to indicate which patient organization you are a member of.”

Answer coding:

1 = I am a member of:

+ Open text input

-1 = Prefer not to say

-9 = Not answered
