# Supplementary material for: Between willingness and practice: a nationwide survey of 1,334 German patient organization members on user involvement in digital service development
Source: Front Digit Health. 2025 Jun 6;7:1591981. doi: 10.3389/fdgth.2025.1591981 (PMC12179215; doi:10.3389/fdgth.2025.1591981)
Supplement: Supplementary file 2 [file Datasheet2.pdf]

Supplementary Material B

B1: Project Advisory Board Composition

The project advisory board of PO representatives was established within the PANDORA research consortium to ensure that the perspectives of patient organizations were integrated into key research steps. Members of the board included representatives from:

- Bundesselbsthilfeverband für Osteoporose e.V.
- Frauenselbsthilfe Krebs Bundesverband e.V.
- Morbus Wilson e.V.
- Mukoviszidose e.V.
- PRO RETINA Deutschland e.V. / Pro Retina — Stiftung zur Verhütung von Blindheit

B2: Umbrella Organizations Used for the Compilation of POs

The membership lists of the following two umbrella organizations were used:

- German National Association for Self-Help (Bundesarbeitsgemeinschaft Selbsthilfe von Menschen mit Behinderung, chronischer Erkrankung und ihren Angehörigen e.V. (BAG SELBSTHILFE))
- Alliance for Chronic Rare Diseases (Allianz Chronischer Seltener Erkrankungen e.V. (ACHSE))

B3: Database Used for the Compilation of POs

The database used for the compilation was the "GRÜNEN ADRESSEN" (GREEN ADDRESSES), maintained by the National Contact and Information Point for Self-Help (Nationale Kontakt- und Informationsstelle zur Anregung und Unterstützung von Selbsthilfegruppen (NAKOS)).

B4: Notes on Variation in Response Counts Across Statements Regarding Conditions and Methods of Involvement

The total number of responses varies slightly between statements, as not all participants responded to each specific statement. Percentages reported in the text are calculated based on the number of respondents for each statement, which are explicitly shown in Figures 1 and 2 for further clarification.
