# Supplementary material for: Between willingness and practice: a nationwide survey of 1,334 German patient organization members on user involvement in digital service development
Source: Front Digit Health. 2025 Jun 6;7:1591981. doi: 10.3389/fdgth.2025.1591981 (PMC12179215; doi:10.3389/fdgth.2025.1591981)
Supplement: Supplementary file 3 [file Datasheet3.pdf]

Supplementary Material C

Table 1. Volunteer Activities Undertaken by PO Members (n = 491).[a]

Volunteer activity	n (%)[b]
Leader or spokesperson of a support group	254 (51.7)
Member of a regional working group, committee or forum	155 (31.6)
Member of a national working group, committee or forum	102 (20.8)
Advisor for other members and affected individuals.	231 (47)
Research partner (e.g., involvement in studies)	77 (15.7)
Navigator/Guide	22 (4.5)
Consultant	90 (18.3)
Spokesperson	58 (11.8)
Treasurer	49 (10)
Other activities	150 (30.5)

[a] The sample size reflects filtering processes within the online survey. Details on these filtering mechanisms can be found in the questionnaire (see Supplementary Material A).

[b] Multiple answers were possible.

Table 2. Types of Digital PO Services Developed with Member Involvement (n = 196).[a]

Type of digital PO service	n (%)[b]
Website	158 (80.6)
App	25 (12.8)
Digital patient registry	12 (6.1)
Digital newsletter	49 (25)
Other	55 (28.1)

[a] The sample size reflects filtering processes within the online survey. Details on these filtering mechanisms can be found in the questionnaire (see Supplementary Material A).

[b] Multiple answers were possible.

Table 3. Members' Involvement in Specific Activities in the Design and Development of Digital PO Services (n = 196).[a]

Type of involvement activity	n (%)[b]
Expressing preferences (e.g., via a survey, interview, group discussion).	108 (55.1)

Type of involvement activity	n (%)[b]
Planning of the digital service (e.g. as a member of a working group).	131 (66.8)
Developing content (texts, videos) for the digital service.	143 (73)
Designing a prototype.	67 (34.2)
Testing and evaluating a prototype.	92 (46.9)
Recommending the digital service to others.	73 (37.2)
Training other members on how to use the digital service.	50 (25.5)
Other activities	21 (10.7)

[a] The sample size reflects filtering processes within the online survey. Details on these filtering mechanisms can be found in the questionnaire (see Supplementary Material A).

[b] Multiple answers were possible.

Table 4. Evaluation of Prior Involvement (n = 196).[a]

Evaluation category	n (%)
Very positive	83 (42.3)
Rather positive	62 (31.6)
Rather negative	26 (13.3)
Very negative	5 (2.6)
Missing[b]	20 (10.2)

[a] The sample size reflects filtering processes within the online survey. Details on these filtering mechanisms can be found in the questionnaire (see Supplementary Material A).

[b] Missing values consist only of ‘prefer not to answer’ responses.

Table 5. Reasons for Refusal to Get Involved (n = 90).[a]

I declined this request because...	n (%)[b]
...did not have the time.	64 (71.1)
...did not see any personal benefit in the digital service.	11 (12.2)
...was not sure if my (technical) skills were sufficient.	36 (40)
...was not sufficiently informed.	7 (7.8)
…had a negative experience with a similar project.	5 (5.6)
Other reasons	26 (28.9)

[a] The sample size reflects filtering processes within the online survey. Details on these filtering mechanisms can be found in the questionnaire (Supplementary Material A).

[b] Multiple answers were possible.

Table 6. Distribution of Importance Ratings for Member Involvement in the Design of New Digital Services (N = 1,334)

How important do you think it is that your patient organization involves members in the design of new digital services?	n (%)[a]
Very important	456 (34.2)
Rather important	627 (47)
Rather unimportant	187 (14)
Not important at all	20 (1.5)
Missing[a]	44 (3.3)

[a] Missing values include 'prefer not to answer' responses and unanswered questions.

Table 7. Distribution of Agreement Ratings on External Collaboration Without Member Involvement (N = 1,334)

I would disapprove of a collaboration if there is no opportunity for me or other members to be involved in the development process.	n (%)[a]
Strongly agree	332 (24.9)
Rather agree	522 (39.1)
Rather disagree	259 (19.4)
Strongly disagree	88 (6.6)
Missing[a]	133 (10)

[a] Missing values include 'prefer not to answer' responses and unanswered questions.

Table 8. Reasons for Lack of Willingness to Get Involved (n = 587).[a]

I would not be interested in getting involved because I...	n (%)[b]
...do not have the time.	363 (61.8)
...do not see any personal benefit in getting involved.	86 (14.7)
...am not sure if my (technical) skills are sufficient.	254 (43.3)
...have had negative experiences with a similar project.	14 (2.4)
...am generally not interested in digital services.	20 (3.4)
Other reasons	110 (18.7)

[a] The sample size reflects filtering processes within the online survey. Details on these filtering mechanisms can be found in the questionnaire (see Supplementary Material A).

[b] Multiple answers were possible.

Table 9. Conditions for Willingness to Get Involved.[a]

I would get involved in the planning and design of a digital service of my patient organization…	Strongly disagree, n (%)	Rather disagree, n (%)	Rather agree, n (%)	Strongly agree, n (%)
...if I feel competent enough to support the tasks at hand. (n = 627)	11 (1.8)	29 (4.6)	303 (48.3)	284 (45.3)
...if the collaboration among all involved is fair and respectful. (n = 625)	11 (1.8)	28 (4.5)	258 (41.3)	328 (52.5)
...even if my role or tasks in the development process are not clearly defined. (n = 621)	85 (13.7)	221 (35.6)	229 (36.9)	86 (13.8)
...even if I have no real influence on the process (e.g., as I would with a say in decisions). (n = 621)	94 (15.1)	228 (36.7)	218 (35.1)	81 (13)
...if I am not required to take on any responsibility. (n = 607)	97 (16)	199 (32.8)	226 (37.2)	85 (14)
...if the project is something I consider relevant or beneficial for myself and the other members. (n = 627)	9 (1.4)	10 (1.6)	278 (44.3)	330 (52.6)
...if the time commitment is as minimal as possible. (n = 616)	37 (6)	205 (33.3)	268 (43.5)	106 (17.2)
...if I am adequately prepared for it (e.g., through training). (n = 617)	41 (6.6)	126 (20.4)	316 (51.2)	134 (21.7)
...even if I receive no financial compensation. (n = 618)	20 (3.2)	47 (7.6)	253 (40.9)	298 (48.2)

[a] These statements were displayed to all respondents who expressed willingness to get involved or did not respond to the willingness question (see Supplementary Material A for details on the filtering mechanisms). However, only those who indicated willingness were included in this analysis (n = 640). Sample sizes vary per statement due to complete case analysis.

Table 10. Members' Willingness to Engage in Different Methods of Involvement.[a]

I could well imagine that I would...	Strongly disagree, n (%)	Rather disagree, n (%)	Rather agree, n (%)	Strongly agree, n (%)
... attend an event organized by my patient organization where members are informed about the development of the digital service. (n = 629)	23 (3.7)	86 (13.7)	269 (42.8)	251 (39.9)
...participate in a survey to share my needs and preferences. (n = 633)	1 (0.2)	5 (0.8)	210 (33.2)	417 (65.9)

I could well imagine that I would...	Strongly disagree, n (%)	Rather disagree, n (%)	Rather agree, n (%)	Strongly agree, n (%)
...advise those responsible (e.g., regarding features or content I find meaningful or important). (n = 622)	37 (5.9)	125 (20.1)	244 (39.2)	216 (34.7)
...try out an initial version (prototype) of the digital service and provide feedback. (n = 634)	15 (2.4)	26 (4.1)	247 (39)	346 (54.6)
...join a member advisory board that regularly exchanges views with those responsible and has a say in decisions. (n = 617)	83 (13.5)	184 (29.8)	198 (32.1)	152 (24.6)
...make all decisions in the development process jointly with those responsible. (n = 618)	105 (17)	167 (27)	193 (31.2)	153 (24.8)

[a] These statements were displayed to all respondents who expressed willingness to get involved or did not respond to the willingness question (see Supplementary Material A for details on the filtering mechanisms). However, only those who indicated willingness were included in this analysis (n = 640). Sample sizes vary per statement due to complete case analysis.

Table 11. Distribution of Sociodemographic and Other Characteristics by Being Approached to Get Involved.

Characteristic	Approached by PO		*p*-value
	Yes, n (%)	No, n (%)	
**Age (n = 1,197)**			0.091[a]
18–29 years (n = 43)	6 (14)	37 (86)	
30–39 years (n = 133)	31 (23.3)	102 (76.7)	
40–49 years (n = 189)	33 (17.5)	156 (82.5)	
50–64 years (n = 527)	135 (25.6)	392 (74.4)	
≥ 65 years (n = 305)	78 (25.6)	227 (74.4)	
**Gender (n = 1,245)**			0.007[b]
Female (n = 851)	180 (21.2)	671 (78.8)	
Male (n = 392)	113 (28.8)	279 (71.2)	
Non-binary or gender diverse (n = 2)	0 (0)	2 (100)	
**Number of indicated diseases and/or disabilities (n = 919)[c]**			0.881[a]
1 (n = 194)	47 (24.2)	147 (75.8)	
2– 3 (n = 419)	91 (21.7)	328 (78.3)	

Characteristic	Approached by PO		*p*-value
	Yes, n (%)	No, n (%)	
4– 5 (n = 197)	47 (23.9)	150 (76.1)	
≥6 (n = 109)	24 (22)	85 (78)	
**Educational attainment (n = 1,235)**			0.171[b]
Basic secondary education certificate *(German: Hauptschulabschluss)* (n = 61)	9 (14.8)	52 (85.2)	
Intermediate secondary education certificate *(German: Realschulabschluss)* (n = 261)	51 (19.5)	210 (80.5)	
Entrance qualification for universities of applied sciences (*German: Fachhochschulreife*) (n = 116)	33 (28.4)	83 (71.6)	
General university entrance qualification (*German: Abitur*) (n = 173)	43 (24.9)	130 (75.1)	
Higher education degree (n = 592)	148 (25)	444 (75)	
No formal qualification (n = 3)	0 (0)	3 (100)	
Other qualification (n = 29)	9 (31)	20 (69)	
**Self-rated digital skills (n = 1,257)**			0.880[b]
Very good (n = 433)	105 (24.2)	328 (75.8)	
Rather good (n = 688)	163 (23.7)	525 (76.3)	
Rather poor (n = 122)	27 (22.1)	95 (77.9)	
Very poor (n = 14)	2 (14.3)	12 (85.7)	
**Membership Background (n = 1,266)**			**<0.001**[a] *
Affected individual (by disease or disability) (n = 952)	214 (22.5)	738 (77.5)	
Relative of an affected individual (n = 238)	52 (21.8)	186 (78.2)	
Other (n = 76)	32 (42.1)	44 (57.9)	
**Duration of Membership (n = 1,269)**			**<0.001**[a] *
Less than 1 year (n = 113)	6 (5.3)	107 (94.7)	
1–2 years (n = 153)	26 (17)	127 (83)	
3–5 years (n = 294)	64 (21.8)	230 (78.2)	
6–10 years (n = 246)	49 (19.9)	197 (80.1)	
More than 10 years (n = 463)	154 (33.3)	309 (66.7)	

Characteristic	Approached by PO		*p*-value
	Yes, n (%)	No, n (%)	
**Voluntary work in PO (n = 1,261)**			**<0.001**[a] *
Yes (n = 468)	207 (44.2)	261 (55.8)	
No (n = 793)	92 (11.6)	701 (88.4)	

[a] *p*-values were computed using the chi-squared test.

[b] *p*-values were computed using the Fisher-Freeman-Halton exact test because more than 20% of the expected cell frequencies were below 5 or the minimum expected frequency was below 1, making the chi-square test inappropriate.

[c] See the footnote to Table 2 in the main text for details on this variable.

* $p < 0.006$ indicates statistical significance after Bonferroni correction for multiple comparisons (8 tests).

Table 12. Distribution of Sociodemographic and Other Characteristics by Agreeing to Get Involved.

Characteristic	Agreeing to get involved		*p*-value
	Yes, n (%)	No, n (%)	
**Age (n = 272)**			0.595[b]
18–29 years (n = 6)	5 (83.3)	1 (16.7)	
30–39 years (n = 31)	21 (67.7)	10 (32.3)	
40–49 years (n = 31)	23 (74.2)	8 (25.8)	
50–64 years (n = 131)	93 (71)	38 (29)	
≥ 65 years (n = 73)	45 (61.6)	28 (38.4)	
**Gender (n = 281)**			0.047[a]
Female (n = 174)	112 (64.4)	62 (35.6)	
Male (n = 107)	81 (75.7)	26 (24.3)	
Non-binary or gender diverse	n/a	n/a	
**Number of indicated diseases and/or disabilities (n = 198)**			0.110[a]
1 (n = 46)	31 (67.4)	15 (32.6)	
2-3 (n = 87)	64 (73.6)	23 (26.4)	
4-5 (n = 41)	24 (58.5)	17 (41.5)	
≥6 (n = 24)	12 (50)	12 (50)	
**Educational attainment (n = 283)**			**<0.001**[a] *
Basic secondary education certificate *(German: Hauptschulabschluss)* (n = 9)	3 (33.3)	6 (66.7)	
Intermediate secondary education certificate *(German: Realschulabschluss)* (n = 47)	18 (38.3)	29 (61.7)	

Characteristic	Agreeing to get involved		*p*-value
	Yes, n (%)	No, n (%)	
Entrance qualification for universities of applied sciences *(German: Fachhochschulreife)* (n = 32)	20 (62.5)	12 (37.5)	
General university entrance qualification *(German: Abitur)* (n = 43)	35 (81.4)	8 (18.6)	
Higher education degree (n = 143)	111 (77.6)	32 (22.4)	
No formal qualification	n/a	n/a	
Other qualification (n = 9)	8 (88.9)	1 (11.1)	
**Self-rated digital skills (n = 285)**			**<0.001**[b] *
Very good (n = 104)	86 (82.7)	18 (17.3)	
Rather good (n = 153)	98 (64.1)	55 (35.9)	
Rather poor (n = 26)	10 (38.5)	16 (61.5)	
Very poor (n = 2)	1 (50)	1 (50)	
**Membership Background (n = 285)**			0.049[a]
Affected individual (by disease or disability) (n = 202)	133 (65.8)	69 (34.2)	
Relative of an affected individual (n = 51)	35 (68.6)	16 (31.4)	
Other (n = 32)	28 (87.5)	4 (12.5)	
**Duration of Membership (n = 286)**			0.673[b]
Less than 1 year (n = 6)	4 (66.7)	2 (33.3)	
1– 2 years (n = 24)	17 (70.8)	7 (29.2)	
3– 5 years (n = 61)	37 (60.7)	24 (39.3)	
6– 10 years (n = 47)	33 (70.2)	14 (29.8)	
More than 10 years (n = 148)	105 (70.9)	43 (29.1)	
**Voluntary work in PO (n = 286)**			**<0.001**[a] *
Yes (n = 200)	161 (80.5)	39 (19.5)	
No (n = 86)	35 (40.7)	51 (59.3)	

[a] *p*-values were computed using the chi-squared test.

[b] *p*-values were computed using the Fisher-Freeman-Halton exact test because more than 20% of the expected cell frequencies were below 5 or the minimum expected frequency was below 1, making the chi-square test inappropriate.

* $p < 0.006$ indicates statistical significance after Bonferroni correction for multiple comparisons (8 tests).

n/a indicates that no responses were observed for this specific variable in the respective category.

Table 13. Distribution of Sociodemographic and Other Characteristics by Willingness to Get Involved.[±]

Characteristic	Willing to get involved		*p*-value
	Yes, n (%)	No, n (%)	
**Age (n = 1,162)**			0.007[a]
18– 29 years (n = 43)	25 (58.1)	18 (41.9)	
30– 39 years (n = 126)	76 (60.3)	50 (39.7)	
40– 49 years (n = 178)	99 (55.6)	79 (44.4)	
50– 64 years (n = 524)	278 (53.1)	246 (46.9)	
≥ 65 years (n = 291)	126 (43.3)	165 (56.7)	
**Gender (n = 1,204)**			**<0.001**[a] *
Female (n = 830)	402 (48.4)	428 (51.6)	
Male (n = 374)	225 (60.2)	149 (39.8)	
Non-binary or gender diverse	n/a	n/a	
**Number of indicated diseases and/or disabilities (n = 890)**			0.352[a]
1 (n = 189)	95 (50.3)	94 (49.7)	
2-3 (n = 406)	213 (52.5)	193 (47.5)	
4-5 (n = 186)	107 (57.5)	79 (42.5)	
≥6 (n = 109)	52 (47.7)	57 (52.3)	
**Educational attainment (n = 1,195)**			**<0.001**[c] *
Basic secondary education certificate *(German: Hauptschulabschluss)* (n = 55)	19 (34.5)	36 (65.5)	
Intermediate secondary education certificate *(German: Realschulabschluss)* (n = 251)	98 (39)	153 (61)	
Entrance qualification for universities of applied sciences *(German: Fachhochschulreife)* (n = 110)	64 (58.2)	46 (41.8)	
General university entrance qualification *(German: Abitur)* (n = 168)	92 (54.8)	76 (45.2)	
Higher education degree(n = 581)	338 (58.2)	243 (41.8)	
No formal qualification (n = 2)	1 (50)	1 (50)	
Other qualification (n = 28)	17 (60.7)	11 (39.3)	
**Self-rated digital skills (n = 1,213)**			**<0.001**[a] *
Very good (n = 426)	271 (63.6)	155 (36.4)	

Characteristic	Willing to get involved		*p*-value
	Yes, n (%)	No, n (%)	
Rather good (n = 659)	330 (50.1)	329 (49.9)	
Rather poor (n = 114)	32 (28.1)	82 (71.9)	
Very poor (n = 14)	2 (14.3)	12 (85.7)	
**Membership background (n = 1,223)**			0.037[a]
Affected individual (by disease or disability) (n = 925)	480 (51.9)	445 (48.1)	
Relative of an affected individual (n = 227)	111 (48.9)	116 (51.1)	
Other (n = 71)	47 (66.2)	24 (33.8)	
**Duration of Membership (n = 1,227)**			0.543[a]
Less than 1 year (n = 108)	64 (59.3)	44 (40.7)	
1–2 years (n = 146)	77 (52.7)	69 (47.3)	
3–5 years (n = 291)	145 (49.8)	146 (50.2)	
6–10 years (n = 237)	120 (50.6)	117 (49.4)	
More than 10 years (n = 445)	234 (52.6)	211 (47.4)	
**Voluntary work in PO (n = 1,219)**			**<0.001**[a] *
Yes (n = 453)	299 (66)	154 (34)	
No (n = 766)	337 (44)	429 (56)	
**Evaluation of prior involvement (n = 170)[d]**			0.408[b]
Very positive (n = 81)	76 (93.8)	5 (6.2)	
Rather positive (n = 59)	53 (89.8)	6 (10.2)	
Rather negative (n = 25)	21 (84)	4 (16)	
Very negative (n = 5)	5 (100)	0 (0)	

[a] *p*-values were computed using the chi-squared test.

[b] *p*-values were computed using the Fisher-Freeman-Halton exact test because more than 20% of the expected cell frequencies were below 5 or the minimum expected frequency was below 1, making the chi-square test inappropriate.

[c] *p*-values were computed using the Fisher-Freeman-Halton exact test (see footnote a for justification). Due to memory limitations in SPSS, the Fisher-Freeman-Halton exact test was calculated using the Monte Carlo method.

[d] This variable was not included in the analyses shown in Table 11 and Table 12, as it relates to prior involvement experiences and therefore cannot be analyzed in relation to whether participants were approached to get involved (Table 11) or agreed to get involved (Table 12). As only respondents who had agreed to such an involvement request were asked to rate their experience, the sample size for this variable is smaller compared to other variables in this analysis. Furthermore, this variable was excluded from the logistic regression analysis to avoid an unnecessary increase in missing cases, which could not be addressed through imputation, as participants were intentionally excluded due to lacking involvement experiences.

* $p < 0.006$ indicates statistical significance after Bonferroni correction for multiple comparisons (9 tests).

n/a indicates that no responses were observed for this specific variable in the respective category.

Table 14. Willingness to Attend an Event on the Development of Digital Services by the PO.±

Characteristic	*I could well imagine that I would...* ...attend an event organized by my patient organization where members are informed about the development of the digital service.				
	Strongly disagree, n (%)	Rather disagree, n (%)	Rather agree, n (%)	Strongly agree, n (%)	*p*-value
**Age (n = 595)**					0.051[a]
18–29 years (n = 25)	2 (8)	2 (8)	13 (52)	8 (32)	
30–39 years (n = 76)	5 (6.6)	18 (23.7)	22 (28.9)	31 (40.8)	
40–49 years (n = 96)	2 (2.1)	15 (15.6)	48 (50)	31 (32.3)	
50–64 years (n = 275)	11 (4)	34 (12.4)	119 (43.3)	111 (40.4)	
65 years and older (n = 123)	3 (2.4)	12 (9.8)	50 (40.7)	58 (47.2)	
**Gender (n = 618)**					**0.001**[b] *
Female (n = 396)	19 (4.8)	67 (16.9)	159 (40.2)	151 (38.1)	
Male (n = 222)	4 (1.8)	16 (7.2)	106 (47.7)	96 (43.2)	
Non-binary or gender diverse	n/a	n/a	n/a	n/a	
**Number of indicated diseases and/or disabilities (n = 461)**					0.024[b]
1 (n = 95)	2 (2.1)	6 (6.3)	44 (46.3)	43 (45.3)	
2-3 (n = 211)	10 (4.7)	23 (10.9)	97 (46)	81 (38.4)	
4-5 (n = 103)	5 (4.9)	15 (14.6)	35 (34)	48 (46.6)	
≥6 (n = 52)	2 (3.8)	14 (26.9)	17 (32.7)	19 (36.5)	

Characteristic	*I could well imagine that I would...* ...attend an event organized by my patient organization where members are informed about the development of the digital service.				
	**Strongly disagree, n (%)**	**Rather disagree, n (%)**	**Rather agree, n (%)**	**Strongly agree, n (%)**	***p*-value**
**Educational attainment (n = 620)**					**<0.001**[a] *****
Basic secondary education certificate *(German: Hauptschulabschluss)* (n = 18)	0 (0)	4 (22.2)	12 (66.7)	2 (11.1)	
Intermediate secondary education certificate *(German: Realschulabschluss)* (n = 94)	6 (6.4)	14 (14.9)	48 (51.1)	26 (27.7)	
Entrance qualification for universities of applied sciences *(German: Fachhochschulreife)* (n = 64)	4 (6.3)	15 (23.4)	27 (42.2)	18 (28.1)	
General university entrance qualification *(German: Abitur)* (n = 92)	2 (2.2)	12 (13)	34 (37)	44 (47.8)	
Higher education degree (n = 334)	9 (2.7)	39 (11.7)	138 (41.3)	148 (44.3)	
No formal qualification (n = 1)	0 (0)	1 (100)	0 (0)	0 (0)	
Other qualification (n = 17)	2 (11.8)	0 (0)	6 (35.3)	9 (52.9)	
**Self-rated digital skills (n = 626)**					0.021[c]
Very good (n = 267)	6 (2.2)	32 (12)	100 (37.5)	129 (48.3)	
Rather good (n = 325)	16 (4.9)	50 (15.4)	153 (47.1)	106 (32.6)	
Rather poor (n = 32)	1 (3.1)	4 (12.5)	13 (40.6)	14 (43.8)	
Very poor (n = 2)	0 (0)	0 (0)	1 (50)	1 (50)	

Characteristic	*I could well imagine that I would...* **...attend an event organized by my patient organization where members are informed about the development of the digital service.**				
	**Strongly disagree, n (%)**	**Rather disagree, n (%)**	**Rather agree, n (%)**	**Strongly agree, n (%)**	***p*-value**
**Membership background (n = 627)**					0.084[b]
Affected individual (by disease or disability) (n = 472)	20 (4.2)	59 (12.5)	197 (41.7)	196 (41.5)	
Relative of an affected individual (n = 109)	2 (1.8)	23 (21.1)	49 (45)	35 (32.1)	
Other (n = 46)	1 (2.2)	3 (6.5)	23 (50)	19 (41.3)	
**Membership duration (n = 629)**					0.058[b]
Less than 1 year (n = 62)	4 (6.5)	16 (25.8)	30 (48.4)	12 (19.4)	
1–2 years (n = 77)	2 (2.6)	10 (13)	35 (45.5)	30 (39)	
3–5 years (n = 143)	7 (4.9)	18 (12.6)	56 (39.2)	62 (43.4)	
6–10 years (n = 118)	5 (4.2)	16 (13.6)	46 (39)	51 (43.2)	
More than 10 years (n = 229)	5 (2.2)	26 (11.4)	102 (44.5)	96 (41.9)	
**Voluntary work in PO (n = 625)**					**<0.001**[b] *
Yes (n = 291)	4 (1.4)	19 (6.5)	110 (37.8)	158 (54.3)	
No (n = 334)	19 (5.7)	66 (19.8)	157 (47)	92 (27.5)	
**Evaluation of prior involvement (n = 154)**					0.074[c]
Very positive (n = 75)	1 (1.3)	2 (2.7)	20 (26.7)	52 (69.3)	
Rather positive (n = 53)	1 (1.9)	1 (1.9)	28 (52.8)	23 (43.4)	
Rather negative (n = 21)	0 (0)	1 (4.8)	6 (28.6)	14 (66.7)	

Characteristic	*I could well imagine that I would...* **...attend an event organized by my patient organization where members are informed about the development of the digital service.**				
	**Strongly disagree, n (%)**	**Rather disagree, n (%)**	**Rather agree, n (%)**	**Strongly agree, n (%)**	***p*-value**
Very negative (n = 5)	0 (0)	0 (0)	3 (60)	2 (40)	

[±] These statements were displayed to all respondents who expressed willingness to get involved or did not respond to the willingness question (see Supplementary Material A for details on the filtering mechanisms). However, only those who indicated willingness were included in this analysis (n = 640). Sample sizes vary due to complete case analysis and, for some variables, because of additional filtering (see Supplementary Material A).

[a] *p*-values were computed using the Fisher-Freeman-Halton exact test because more than 20% of the expected cell frequencies were below 5 or the minimum expected frequency was below 1, making the chi-square test inappropriate. Due to memory limitations in SPSS, the Fisher-Freeman-Halton exact test was calculated using the Monte Carlo method.

[b] *p*-values were computed using the chi-squared test.

[c] *p*-values were computed using the Fisher-Freeman-Halton exact test (see footnote a for justification).

* $p < 0.006$ indicates statistical significance after Bonferroni correction for multiple comparisons (9 tests).

n/a indicates that no responses were observed for this specific variable in the respective category

Table 15. Willingness to Participate in a Survey on Needs and Preferences.[±]

Characteristic	*I could well imagine that I would...* **...participate in a survey to share my needs and preferences.**				
	**Strongly disagree, n (%)**	**Rather disagree, n (%)**	**Rather agree, n (%)**	**Strongly agree, n (%)**	***p*-value**
**Age (n = 599)**					0.015[a]
18–29 years (n = 25)	0 (0)	0 (0)	5 (20)	20 (80)	
30–39 years (n = 76)	0 (0)	0 (0)	12 (15.8)	64 (84.2)	
40–49 years (n = 98)	0 (0)	1 (1)	31 (31.6)	66 (67.3)	

Characteristic	*I could well imagine that I would...* **...participate in a survey to share my needs and preferences.**				
	**Strongly disagree, n (%)**	**Rather disagree, n (%)**	**Rather agree, n (%)**	**Strongly agree, n (%)**	***p*-value**
50–64 years (n = 275)	1 (0.4)	3 (1.1)	103 (37.5)	168 (61.1)	
65 years and older (n = 125)	0 (0)	1 (0.8)	47 (37.6)	77 (61.6)	
**Gender (n = 622)**					0.314[c]
Female (n = 400)	1 (0.3)	2 (0.5)	126 (31.5)	271 (67.8)	
Male (n = 222)	0 (0)	3 (1.4)	80 (36)	139 (62.6)	
Non-binary or gender diverse	n/a	n/a	n/a	n/a	
**Number of indicated diseases and/or disabilities (n = 463)**					0.257[c]
1 (n = 95)	0 (0)	1 (1.19	29 (30.5)	65 (68.4)	
2-3 (n = 211)	0 (0)	1 (0.5)	79 (37.4)	131 (62.1)	
4-5 (n = 105)	0 (0)	0 (0)	28 (26.7)	77 (73.3)	
≥6 (n = 52)	0 (0)	1 (1.9)	18 (34.6)	33 (63.5)	
**Educational attainment (n = 624)**					0.037[a]
Basic secondary education certificate *(German: Hauptschulabschluss)* (n = 19)	0 (0)	0 (0)	12 (63.2)	7 (36.8)	
Intermediate secondary education certificate *(German: Realschulabschluss)* (n = 94)	0 (0)	2 (2.1)	36 (38.3)	56 (59.6)	
Entrance qualification for universities of applied sciences *(German: Fachhochschulreife)* (n = 64)	1 (1.6)	0 (0)	25 (39.1)	38 (59.4)	

Characteristic	*I could well imagine that I would...* **...participate in a survey to share my needs and preferences.**				
	**Strongly disagree, n (%)**	**Rather disagree, n (%)**	**Rather agree, n (%)**	**Strongly agree, n (%)**	***p*-value**
General university entrance qualification *(German: Abitur)* (n = 92)	0 (0)	1 (1.1)	23 (25)	68 (73.9)	
Higher education degree (n = 337)	0 (0)	2 (0.6)	105 (31.2)	230 (68.2)	
No formal qualification (n = 1)	0 (0)	0 (0)	0 (0)	1 (100)	
Other qualification (n = 17)	0 (0)	0 (0)	7 (41.2)	10 (58.8)	
**Self-rated digital skills (n = 629)**					**<0.001**[c] *
Very good (n = 268)	0 (0)	2 (0.7)	61 (22.8)	205 (76.5)	
Rather good (n = 327)	1 (0.3)	3 (0.9)	133 (40.79	190 (58.1)	
Rather poor (n = 32)	0 (0)	0 (0)	15 (46.9)	17 (53.1)	
Very poor (n = 2)	0 (0)	0 (0)	1 (50)	1 (50)	
**Membership background (n = 631)**					0.149[c]
Affected individual (by disease or disability) (n = 474)	0 (0)	3 (0.6)	157 (33.1)	314 (66.2)	
Relative of an affected individual (n = 111)	0 (0)	1 (0.9)	35 (31.5)	75 (67.6)	
Other (n = 46)	1 (2.2)	1 (2.2)	17 (37)	27 (58.7)	
**Membership duration (n = 633)**					0.652[c]
Less than 1 year (n = 63)	0 (0)	1 (1.6)	24 (38.1)	38 (60.3)	
1–2 years (n = 77)	0 (0)	1 (1.3)	24 (31.2)	52 (67.5)	
3–5 years (n = 144)	1 (0.7)	0 (0)	41 (28.5)	102 (70.8)	

Characteristic	*I could well imagine that I would...* **...participate in a survey to share my needs and preferences.**				
	**Strongly disagree, n (%)**	**Rather disagree, n (%)**	**Rather agree, n (%)**	**Strongly agree, n (%)**	***p*-value**
6–10 years (n = 119)	0 (0)	1 (0.8)	40 (33.6)	78 (65.5)	
More than 10 years (n = 230)	0 (0)	2 (0.9)	81 (35.2)	147 63.9)	
**Voluntary work in PO (n = 629)**					0.206[c]
Yes (n = 292)	1 (0.3)	4 (1.4)	91 (31.2)	196 (67.1)	
No (n = 337)	0 (0)	1 (0.3)	118 (35)	218 (64.7)	
**Evaluation of prior involvement (n = 154)**					0.987[c]
Very positive (n = 75)	0 (0)	1 (1.3)	19 (25.3)	55 (73.3)	
Rather positive (n = 53)	0 (0)	0 (0)	13 (24.5)	40 (75.5)	
Rather negative (n = 21)	0 (0)	0 (0)	6 (28.6)	15 (71.4)	
Very negative (n = 5)	0 (0)	0 (0)	1 (20)	4 (80)	

± These statements were displayed to all respondents who expressed willingness to get involved or did not respond to the willingness question (see Supplementary Material A for details on the filtering mechanisms). However, only those who indicated willingness were included in this analysis (n = 640). Sample sizes vary due to complete case analysis and, for some variables, because of additional filtering (see Supplementary Material A).

[a] *p*-values were computed using the Fisher-Freeman-Halton exact test because more than 20% of the expected cell frequencies were below 5 or the minimum expected frequency was below 1, making the chi-square test inappropriate. Due to memory limitations in SPSS, the Fisher-Freeman-Halton exact test was calculated using the Monte Carlo method.

[b] *p*-values were computed using the chi-squared test.

[c] *p*-values were computed using the Fisher-Freeman-Halton exact test (see footnote a for justification).

* $p < 0.006$ indicates statistical significance after Bonferroni correction for multiple comparisons (9 tests).

n/a indicates that no responses were observed for this specific variable in the respective category

Table 16. Willingness to Provide Advice to Those Responsible for Developing the Digital Service.±

Characteristic	*I could well imagine that I would...* ...advise those responsible (e.g., regarding features or content I find meaningful or important).				
	Strongly disagree, n (%)	Rather disagree, n (%)	Rather agree, n (%)	Strongly agree, n (%)	*p*-value
**Age (n = 591)**					0.259[b]
18–29 years (n = 25)	1 (4)	8 (32)	10 (40)	6 (24)	
30–39 years (n = 75)	4 (5.3)	14 (18.7)	35 (46.7)	22 (29.3)	
40–49 years (n = 97)	3 (3.1)	14 (14.4)	47 (48.5)	33 (34)	
50–64 years (n = 272)	20 (7.4)	61 (22.4)	93 (34.2)	98 (36)	
65 years and older (n = 122)	5 (4.1)	21 (17.2)	50 (41)	46 (37.7)	
**Gender (n = 611)**					0.026[b]
Female (n = 391)	30 (7.7)	83 (21.2)	147 (37.6)	131 (33.5)	
Male (n = 220)	5 (2.3)	40 (18.2)	95 (43.2)	80 (36.4)	
Non-binary or gender diverse	n/a	n/a	n/a	n/a	
**Number of indicated diseases and/or disabilities (n = 456)**					0.062[b]
1 (n = 93)	2 (2.2)	19 (20.4)	44 (47.3)	28 (30.1)	
2-3 (n = 207)	9 (4.3)	42 (20.3)	86 (41.5)	70 (33.8)	
4-5 (n = 104)	11 (10.6)	16 (15.4)	34 (32.7)	43 (41.3)	
≥6 (n = 52)	6 (11.5)	10 (19.2)	16 (30.8)	20 (38.5)	

Characteristic	*I could well imagine that I would...* **...advise those responsible (e.g., regarding features or content I find meaningful or important).**				
	**Strongly disagree, n (%)**	**Rather disagree, n (%)**	**Rather agree, n (%)**	**Strongly agree, n (%)**	***p*-value**
**Educational attainment (n = 613)**					**0.004**[a] *
Basic secondary education certificate *(German: Hauptschulabschluss)* (n = 19)	2 (10.5)	7 (36.8)	7 (36.8)	3 (15.8)	
Intermediate secondary education certificate *(German: Realschulabschluss)* (n = 93)	8 (8.6)	25 (26.9)	42 (45.2)	18 (19.4)	
Entrance qualification for universities of applied sciences *(German: Fachhochschulreife)* (n = 64)	7 (10.9)	14 (21.9)	25 (39.1)	18 (28.1)	
General university entrance qualification *(German: Abitur)* (n = 92)	6 (6.5)	20 (21.7)	27 (29.3)	39 (42.4)	
Higher education degree (n = 328)	11 (3.4)	55 (16.8)	133 (40.5)	129 (39.3)	
No formal qualification (n = 1)	0 (0)	0 (0)	1 (100)	0 (0)	
Other qualification (n = 16)	1 (6.3)	3 (18.8)	7 (43.8)	5 (31.3)	
**Self-rated digital skills (n = 618)**					**<0.001**[a] *
Very good (n = 267)	7 (2.6)	46 (17.2)	97 (36.3)	117 (43.8)	
Rather good (n = 317)	27 (8.5)	72 (22.7)	133 (42)	85 (26.8)	
Rather poor (n = 32)	3 (9.4)	5 (15.6)	13 (40.6)	11 (34.4)	
Very poor (n = 2)	0 (0)	1 (50)	0 (0)	1 (50)	

Characteristic	*I could well imagine that I would...* ...advise those responsible (e.g., regarding features or content I find meaningful or important).				
	Strongly disagree, n (%)	Rather disagree, n (%)	Rather agree, n (%)	Strongly agree, n (%)	*p*-value
**Membership background (n = 620)**					0.130[b]
Affected individual (by disease or disability) (n = 466)	29 (6.2)	89 (19.1)	183 (39.3)	165 (35.4)	
Relative of an affected individual (n = 108)	7 (6.5)	31 (28.7)	39 (36.1)	31 (28.7)	
Other (n = 46)	1 (2.2)	5 (10.9)	21 (45.7)	19 (41.3)	
**Membership duration (n = 622)**					0.061[b]
Less than 1 year (n = 61)	7 (11.5)	11 (18)	29 (47.5)	14 (23)	
1–2 years (n = 77)	3 (3.9)	17 (22.1)	29 (37.7)	28 (36.4)	
3–5 years (n = 140)	11 (7.9)	36 (25.7)	45 (32.1)	48 (34.3)	
6–10 years (n = 118)	8 (6.8)	27 (22.9)	46 (39)	37 (31.4)	
More than 10 years (n = 226)	8 (3.5)	34 (15)	95 (42)	89 (39.4)	
**Voluntary work in PO (n = 618)**					**<0.001**[b] *
Yes (n = 291)	8 (2.7)	42 (14.4)	105 (36.1)	136 (46.7)	
No (n = 327)	29 (8.9)	82 (25.1)	137 (41.9)	79 (24.2)	
**Evaluation of prior involvement (n = 154)**					0.085[c]
Very positive (n = 75)	1 (1.3)	3 (4)	26 (34.7)	45 (60)	
Rather positive (n = 53)	0 (0)	9 (17)	21 (39.6)	23 (43.4)	
Rather negative (n = 21)	1 (4.8)	2 (9.5)	4 (19)	14 (66.7)	

Characteristic	*I could well imagine that I would...* ...advise those responsible (e.g., regarding features or content I find meaningful or important).				
	Strongly disagree, n (%)	Rather disagree, n (%)	Rather agree, n (%)	Strongly agree, n (%)	*p*-value
Very negative (n = 5)	0 (0)	0 (0)	1 (20)	4 (80)	

[±] These statements were displayed to all respondents who expressed willingness to get involved or did not respond to the willingness question (see Supplementary Material A for details on the filtering mechanisms). However, only those who indicated willingness were included in this analysis (n = 640). Sample sizes vary due to complete case analysis and, for some variables, because of additional filtering (see Supplementary Material A).

[a] *p*-values were computed using the Fisher-Freeman-Halton exact test because more than 20% of the expected cell frequencies were below 5 or the minimum expected frequency was below 1, making the chi-square test inappropriate. Due to memory limitations in SPSS, the Fisher-Freeman-Halton exact test was calculated using the Monte Carlo method.

[b] *p*-values were computed using the chi-squared test.

[c] *p*-values were computed using the Fisher-Freeman-Halton exact test (see footnote a for justification).

* $p < 0.006$ indicates statistical significance after Bonferroni correction for multiple comparisons (9 tests).

n/a indicates that no responses were observed for this specific variable in the respective category

Table 17. Willingness to Test and Provide Feedback on a Prototype of a Digital Service.[±]

Characteristic	*I could well imagine that I would...* ...try out an initial version (prototype) of the digital service and provide feedback.				
	Strongly disagree, n (%)	Rather disagree, n (%)	Rather agree, n (%)	Strongly agree, n (%)	*p*-value
**Age (n = 599)**					0.268[a]
18–29 years (n = 25)	0 (0)	0 (0)	10 (40)	15 (60)	
30–39 years (n = 76)	1 (1.3)	3 (3.9)	20 (26.3)	52 (68.4)	
40–49 years (n = 99)	1 (1)	2 (2)	37 (37.4)	59 (59.6)	

Characteristic	*I could well imagine that I would...*<br>**...try out an initial version (prototype) of the digital service and provide feedback.**				
	**Strongly disagree, n (%)**	**Rather disagree, n (%)**	**Rather agree, n (%)**	**Strongly agree, n (%)**	***p*-value**
50–64 years (n = 274)	9 (3.3)	14 (5.1)	107 (39.1)	144 (52.6)	
65 years and older (n = 125)	4 (3.2)	6 (4.8)	57 (45.6)	58 (46.4)	
**Gender (n = 622)**					0.396[b]
Female (n = 400)	11 (2.8)	14 (3.5)	150 (37.5)	225 (56.3)	
Male (n = 222)	4 (1.8)	12 (5.4)	92 (41.4)	114 (51.4)	
Non-binary or gender diverse	n/a	n/a	n/a	n/a	
**Number of indicated diseases and/or disabilities (n = 463)**					0.397[a]
1 (n = 95)	2 (2.1)	3 (3.2)	34 (35.8)	56 (58.9)	
2-3 (n = 210)	6 (2.9)	7 (3.3)	89 (42.4)	108 (51.4)	
4-5 (n = 106)	1 (0.9)	8 (7.5)	36 (34)	61 (57.5)	
≥6 (n = 52)	2 (3.8)	3 (5.8)	15 (28.8)	32 (61.5)	
**Educational attainment (n = 624)**					**<0.001**[a] *
Basic secondary education certificate *(German: Hauptschulabschluss)* (n = 19)	0 (0)	3 (15.8)	8 (42.1)	8 (42.1)	
Intermediate secondary education certificate *(German: Realschulabschluss)* (n = 94)	7 (7.4)	6 (6.4)	39 (41.5)	42 (44.7)	
Entrance qualification for universities of applied sciences *(German: Fachhochschulreife)* (n = 64)	1 (1.6)	4 (6.3)	27 (42.2)	32 (50)	

Characteristic	*I could well imagine that I would...* **...try out an initial version (prototype) of the digital service and provide feedback.**				
	**Strongly disagree, n (%)**	**Rather disagree, n (%)**	**Rather agree, n (%)**	**Strongly agree, n (%)**	***p*-value**
General university entrance qualification *(German: Abitur)* (n = 92)	0 (0)	2 (2.2)	31 (33.7)	59 (64.1)	
Higher education degree (n = 337)	3 (0.9)	11 (3.3)	130 (38.6)	193 (57.3)	
No formal qualification (n = 1)	0 (0)	0 (0)	1 (100)	0 (0)	
Other qualification (n = 17)	3 (17.6)	0 (0)	7 (41.2)	7 (41.2)	
**Self-rated digital skills (n = 630)**					**<0.001**[a] *****
Very good (n = 270)	2 (0.7)	7 (2.6)	76 (28.1)	185 (68.5)	
Rather good (n = 326)	11 (3.4)	18 (5.5)	149 (45.7)	148 (45.4)	
Rather poor (n = 32)	2 (6.3)	1 (3.1)	17 (53.1)	12 (37.5)	
Very poor (n = 2)	0 (0)	0 (0)	2 (100)	0 (0)	
**Membership background (n = 632)**					0.898[c]
Affected individual (by disease or disability) (n = 475)	12 (2.5)	22 (4.6)	178 (37.5)	263 (55.4)	
Relative of an affected individual (n = 111)	2 (1.8)	3 (2.7)	48 (43.2)	58 (52.3)	
Other (n = 46)	1 (2.2)	1 (2.2)	20 (43.5)	24 (52.2)	
**Membership duration (n = 634)**					0.457[a]
Less than 1 year (n = 64)	3 (4.7)	1 (1.6)	29 (45.3)	31 (48.4)	
1–2 years (n = 77)	1 (1.3)	2 (2.6)	29 (37.7)	45 (58.4)	
3–5 years (n = 144)	3 (2.1)	9 (6.3)	45 (31.3)	87 (60.4)	

Characteristic	*I could well imagine that I would...* **...try out an initial version (prototype) of the digital service and provide feedback.**				
	**Strongly disagree, n (%)**	**Rather disagree, n (%)**	**Rather agree, n (%)**	**Strongly agree, n (%)**	***p*-value**
6–10 years (n = 119)	3 (2.5)	7 (5.9)	48 (40.3)	61 (51.3)	
More than 10 years (n = 230)	5 (2.2)	7 (3)	96 (41.7)	122 (53)	
**Voluntary work in PO (n = 630)**					0.042[b]
Yes (n = 294)	6 (2)	8 (2.7)	103 (35)	177 (60.2)	
No (n = 336)	9 (2.7)	18 (5.4)	142 (42.3)	167 (49.7)	
**Evaluation of prior involvement (n = 154)**					0.612[c]
Very positive (n = 75)	2 (2.7)	0 (0)	20 (26.7)	53 (70.7)	
Rather positive (n = 53)	0 (0)	2 (3.8)	15 (28.3)	36 (67.9)	
Rather negative (n = 21)	0 (0)	1 (4.8)	7 (33.3)	13 (61.9)	
Very negative (n = 5)	0 (0)	0 (0)	1 (20)	4 (80)	

± These statements were displayed to all respondents who expressed willingness to get involved or did not respond to the willingness question (see Supplementary Material A for details on the filtering mechanisms). However, only those who indicated willingness were included in this analysis (n = 640). Sample sizes vary due to complete case analysis and, for some variables, because of additional filtering (see Supplementary Material A).

[a] *p*-values were computed using the Fisher-Freeman-Halton exact test because more than 20% of the expected cell frequencies were below 5 or the minimum expected frequency was below 1, making the chi-square test inappropriate. Due to memory limitations in SPSS, the Fisher-Freeman-Halton exact test was calculated using the Monte Carlo method.

[b] *p*-values were computed using the chi-squared test.

[c] *p*-values were computed using the Fisher-Freeman-Halton exact test (see footnote a for justification).

* $p < 0.006$ indicates statistical significance after Bonferroni correction for multiple comparisons (9 tests).

n/a indicates that no responses were observed for this specific variable in the respective category

Table 18. Willingness to Join a Member Advisory Board.[±]

Characteristic	*I could well imagine that I would...* **...join a member advisory board that regularly exchanges views with those responsible and has a say in decisions.**				
	**Strongly disagree, n (%)**	**Rather disagree, n (%)**	**Rather agree, n (%)**	**Strongly agree, n (%)**	***p*-value**
**Age (n = 584)**					0.724[b]
18–29 years (n = 24)	5 (20.8)	7 (29.2)	11 (45.8)	1 (4.2)	
30–39 years (n = 73)	10 (13.7)	26 (35.6)	19 (26)	18 (24.7)	
40–49 years (n = 95)	11 (11.6)	29 (30.5)	30 (31.6)	25 (26.3)	
50–64 years (n = 270)	34 (12.6)	80 (29.6)	87 (32.2)	69 (25.6)	
65 years and older (n = 122)	15 (12.3)	37 (30.3)	38 (31.1)	32 (26.2)	
**Gender (n = 605)**					0.091[b]
Female (n = 390)	57 (14.6)	124 (31.8)	116 (29.7)	93 (23.8)	
Male (n = 215)	21 (9.8)	57 (26.5)	79 (36.7)	58 (27)	
Non-binary or gender diverse	n/a	n/a	n/a	n/a	
**Number of indicated diseases and/or disabilities (n = 454)**					0.034[b]
1 (n = 94)	7 (7.4)	31 (33)	33 (35.1)	23 (24.5)	
2-3 (n = 203)	27 (13.3)	67 (33)	65 (32)	44 (21.7)	
4-5 (n = 105)	19 (18.1)	20 (19)	31 (29.5)	35 (33.3)	
≥6 (n = 52)	11 (21.2)	11 (21.2)	20 (38.5)	10 (19.2)	

Characteristic	*I could well imagine that I would...* **...join a member advisory board that regularly exchanges views with those responsible and has a say in decisions.**				
	**Strongly disagree, n (%)**	**Rather disagree, n (%)**	**Rather agree, n (%)**	**Strongly agree, n (%)**	***p*-value**
**Educational attainment (n = 607)**					**<0.001**[a] *****
Basic secondary education certificate *(German: Hauptschulabschluss)* (n = 19)	3 (15.8)	10 (52.6)	6 (31.6)	0 (0)	
Intermediate secondary education certificate *(German: Realschulabschluss)* (n = 92)	14 (15.2)	35 (38)	31 (33.7)	12 (13)	
Entrance qualification for universities of applied sciences *(German: Fachhochschulreife)* (n = 63)	10 (15.9)	21 (33.3)	18 (28.6)	14 (22.2)	
General university entrance qualification *(German: Abitur)* (n = 91)	15 (16.5)	25 (27.5)	25 (27.5)	26 (28.6)	
Higher education degree (n = 326)	32 (9.8)	88 (27)	110 (33.7)	96 (29.4)	
No formal qualification (n = 1)	0 (0)	1 (100)	0 (0)	0 (0)	
Other qualification (n = 15)	6 (40)	2 (13.3)	5 (33.3)	2 (13.3)	
**Self-rated digital skills (n = 613)**					0.007[a]
Very good (n = 265)	30 (11.3)	62 (23.4)	88 (33.2)	85 (32.1)	
Rather good (n = 316)	48 (15.2)	108 (34.2)	99 (31.3)	61 (19.3)	
Rather poor (n = 30)	4 (13.3)	12 (40)	9 (30)	5 (16.7)	
Very poor (n = 2)	0 (0)	1 (50)	1 (50)	0 (0)	

Characteristic	*I could well imagine that I would...* **...join a member advisory board that regularly exchanges views with those responsible and has a say in decisions.**				
	**Strongly disagree, n (%)**	**Rather disagree, n (%)**	**Rather agree, n (%)**	**Strongly agree, n (%)**	***p*-value**
**Membership background (n = 615)**					0.617[b]
Affected individual (by disease or disability) (n = 464)	66 (14.2)	133 (28.7)	151 (32.5)	114 (24.6)	
Relative of an affected individual (n = 107)	13 (12.1)	39 (36.4)	30 (28)	25 (23.4)	
Other (n = 44)	4 (9.1)	11 (25)	17 (38.6)	12 (27.3)	
**Membership duration (n = 617)**					0.415[b]
Less than 1 year (n = 62)	12 (19.4)	23 (37.1)	21 (33.9)	6 (9.7)	
1–2 years (n = 75)	10 (13.3)	23 (30.7)	23 (30.7)	19 (25.3)	
3–5 years (n = 140)	16 (11.4)	45 (32.1)	39 (27.9)	40 (28.6)	
6–10 years (n = 119)	17 (14.3)	34 (28.6)	39 (32.8)	29 (24.4)	
More than 10 years (n = 221)	28 (12.7)	59 (26.7)	76 (34.4)	58 (26.2)	
**Voluntary work in PO (n = 613)**					**<0.001**[b] *
Yes (n = 286)	20 (7)	59 (20.6)	101 (35.3)	106 (37.1)	
No (n = 327)	62 (19)	123 (37.6)	96 (29.4)	46 (14.1)	
**Evaluation of prior involvement (n = 149)**					0.343[c]
Very positive (n = 71)	5 (7)	6 (8.5)	27 (38)	33 (46.5)	
Rather positive (n = 52)	4 (7.7)	12 (23.1)	17 (32.7)	19 (36.5)	
Rather negative (n = 21)	1 (4.8)	4 (19)	8 (38.1)	8 (38.1)	

Characteristic	*I could well imagine that I would...* **...join a member advisory board that regularly exchanges views with those responsible and has a say in decisions.**				
	**Strongly disagree, n (%)**	**Rather disagree, n (%)**	**Rather agree, n (%)**	**Strongly agree, n (%)**	***p*-value**
Very negative (n = 5)	0 (0)	1 (20)	0 (0)	4 (80)	

[±] These statements were displayed to all respondents who expressed willingness to get involved or did not respond to the willingness question (see Supplementary Material A for details on the filtering mechanisms). However, only those who indicated willingness were included in this analysis (n = 640). Sample sizes vary due to complete case analysis and, for some variables, because of additional filtering (see Supplementary Material A).

[a] *p*-values were computed using the Fisher-Freeman-Halton exact test because more than 20% of the expected cell frequencies were below 5 or the minimum expected frequency was below 1, making the chi-square test inappropriate. Due to memory limitations in SPSS, the Fisher-Freeman-Halton exact test was calculated using the Monte Carlo method.

[b] *p*-values were computed using the chi-squared test.

[c] *p*-values were computed using the Fisher-Freeman-Halton exact test (see footnote a for justification).

* $p < 0.006$ indicates statistical significance after Bonferroni correction for multiple comparisons (9 tests).

n/a indicates that no responses were observed for this specific variable in the respective category

Table 19. Willingness to Make Joint Decisions Throughout the Development Process.[±]

Characteristic	*I could well imagine that I would...* **...make all decisions in the development process jointly with those responsible.**				
	**Strongly disagree, n (%)**	**Rather disagree, n (%)**	**Rather agree, n (%)**	**Strongly agree, n (%)**	***p*-value**
**Age (n = 586)**					0.704[b]
18–29 years (n = 23)	6 (26.1)	7 (30.4)	7 (30.4)	3 (13)	
30–39 years (n = 74)	18 (24.3)	20 (27)	20 (27)	16 (21.6)	
40–49 years (n = 96)	14 (14.6)	30 (31.3)	29 (30.2)	23 (24)	

Characteristic	*I could well imagine that I would...* **...make all decisions in the development process jointly with those responsible.**				
	**Strongly disagree, n (%)**	**Rather disagree, n (%)**	**Rather agree, n (%)**	**Strongly agree, n (%)**	***p*-value**
50–64 years (n = 272)	43 (15.8)	73 (26.8)	84 (30.9)	72 (26.5)	
65 years and older (n = 121)	18 (14.9)	28 (23.1)	43 (35.5)	32 (26.4)	
**Gender (n = 606)**					**<0.001**[b] *
Female (n = 388)	81 (20.9)	109 (28.1)	110 (28.4)	88 (22.7)	
Male (n = 218)	21 (9.6)	54 (24.8)	79 (36.2)	64 (29.4)	
Non-binary or gender diverse	n/a	n/a	n/a	n/a	
**Number of indicated diseases and/or disabilities (n = 456)**					0.006[b]
1 (n = 94)	12 (12.8)	31 (33)	27 (28.7)	24 (25.5)	
2-3 (n = 206)	33 (16)	61 (29.6)	66 (32)	46 (22.3)	
4-5 (n = 104)	25 (24)	17 (16.3)	25 (24)	37 (35.6)	
≥6 (n = 52)	13 (25)	9 (17.3)	21 (40.4)	9 (17.3)	
**Educational attainment (n = 608)**					0.020[a]
Basic secondary education certificate *(German: Hauptschulabschluss)* (n = 18)	4 (22.2)	6 (33.3)	7 (38.9)	1 (5.6)	
Intermediate secondary education certificate *(German: Realschulabschluss)* (n = 92)	21 (22.8)	24 (26.1)	33 (35.9)	14 (15.2)	
Entrance qualification for universities of applied sciences *(German: Fachhochschulreife)* (n = 64)	15 (23.4)	18 (28.1)	18 (28.1)	13 (20.3)	

Characteristic	*I could well imagine that I would...* **...make all decisions in the development process jointly with those responsible.**				
	**Strongly disagree, n (%)**	**Rather disagree, n (%)**	**Rather agree, n (%)**	**Strongly agree, n (%)**	***p*-value**
General university entrance qualification *(German: Abitur)* (n = 92)	16 (17.4)	24 (26.1)	24 (26.1)	28 (30.4)	
Higher education degree (n = 325)	41 (12.6)	90 (27.7)	102 (31.4)	92 (28.3)	
No formal qualification (n = 1)	0 (0)	1 (100)	0 (0)	0 (0)	
Other qualification (n = 16)	7 (43.8)	2 (12.5)	5 (31.3)	2 (12.5)	
**Self-rated digital skills (n = 615)**					0.016[a]
Very good (n = 263)	38 (14.4)	58 (22.1)	82 (31.2)	85 (32.3)	
Rather good (n = 318)	60 (18.9)	97 (30.5)	100 (31.4)	61 (19.2)	
Rather poor (n = 32)	6 (18.8)	11 (34.4)	9 (28.1)	6 (18.8)	
Very poor (n = 2)	0 (0)	1 (50)	1 (50)	0 (0)	
**Membership background (n = 616)**					0.085[b]
Affected individual (by disease or disability) (n = 466)	84 (18)	121 (26)	143 (30.7)	118 (25.3)	
Relative of an affected individual (n = 107)	17 (15.9)	38 (35.5)	32 (29.9)	20 (18.7)	
Other (n = 43)	4 (9.3)	7 (16.3)	18 (41.9)	14 (32.6)	
**Membership duration (n = 618)**					0.125[b]
Less than 1 year (n = 63)	13 (20.6)	25 (39.7)	19 (30.2)	6 (9.5)	
1–2 years (n = 74)	13 (17.6)	20 (27)	23 (31.1)	18 (24.3)	
3–5 years (n = 140)	25 (17.9)	42 (30)	38 (27.1)	35 (25)	

Characteristic	*I could well imagine that I would...* **...make all decisions in the development process jointly with those responsible.**				
	**Strongly disagree, n (%)**	**Rather disagree, n (%)**	**Rather agree, n (%)**	**Strongly agree, n (%)**	***p*-value**
6–10 years (n = 117)	24 (20.5)	27 (23.1)	36 (30.8)	30 (25.6)	
More than 10 years (n = 224)	30 (13.4)	53 (23.7)	77 (34.4)	64 (28.6)	
**Voluntary work in PO (n = 615)**					**<0.001**[b] *
Yes (n = 290)	21 (7.2)	52 (17.9)	107 (36.9)	110 (37.9)	
No (n = 325)	83 (25.5)	114 (35.1)	86 (26.5)	42 (12.9)	
**Evaluation of prior involvement (n = 152)**					0.625[c]
Very positive (n = 73)	3 (4.1)	8 (11)	24 (32.9)	38 (52.1)	
Rather positive (n = 53)	3 (5.7)	7 (13.2)	23 (43.4)	20 (37.7)	
Rather negative (n = 21)	1 (4.8)	2 (9.5)	8 (38.1)	10 (47.6)	
Very negative (n = 5)	0 (0)	1 (20)	0 (0)	4 (80)	

[±] These statements were displayed to all respondents who expressed willingness to get involved or did not respond to the willingness question (see Supplementary Material A for details on the filtering mechanisms). However, only those who indicated willingness were included in this analysis (n = 640). Sample sizes vary due to complete case analysis and, for some variables, because of additional filtering (see Supplementary Material A).

[a] *p*-values were computed using the Fisher-Freeman-Halton exact test because more than 20% of the expected cell frequencies were below 5 or the minimum expected frequency was below 1, making the chi-square test inappropriate. Due to memory limitations in SPSS, the Fisher-Freeman-Halton exact test was calculated using the Monte Carlo method.

[b] *p*-values were computed using the chi-squared test.

[c] *p*-values were computed using the Fisher-Freeman-Halton exact test (see footnote a for justification).

* $p < 0.006$ indicates statistical significance after Bonferroni correction for multiple comparisons (9 tests).

n/a indicates that no responses were observed for this specific variable in the respective category

Table 20. Distribution of Self-Rated Digital Skills Across Age Groups (n = 1,242).±

Age	Self-rated digital skills				
	**Very good, n (%)**	**Rather good, n (%)**	**Rather poor, n (%)**	**Very poor, n (%)**	***p*-value**
18–29 years (n = 44)	28 (63.6)	16 (36.4)	0 (0)	0 (0)	**<0.001**[a] *
30–39 years (n = 136)	75 (55.1)	55 (40.4)	6 (4.4)	0 (0)	
40–49 years (n = 197)	88 (44.7)	103 (52.3)	5 (2.5)	1 (0.5)	
50–64 years (n = 551)	166 (30.1)	326 (59.2)	53 (9.6)	6 (1.1)	
65 years and older (n = 314)	61 (19.4)	189 (60.2)	58 (18.5)	6 (1.9)	

± The sample size is smaller than the total sample due to complete case analysis.

[a] *p*-value was computed using the Fisher-Freeman-Halton exact test because more than 20% of the expected cell frequencies were below 5 or the minimum expected frequency was below 1, making the chi-square test inappropriate. Due to memory limitations in SPSS, the Fisher-Freeman-Halton exact test was calculated using the Monte Carlo method.

* $p < 0.05$ indicates statistical significance.
